# *In vitro* Biochemical Characterization of All Barley Endosperm Starch Synthases

**DOI:** 10.3389/fpls.2015.01265

**Published:** 2016-01-28

**Authors:** Jose A. Cuesta-Seijo, Morten M. Nielsen, Christian Ruzanski, Katarzyna Krucewicz, Sophie R. Beeren, Maja G. Rydhal, Yayoi Yoshimura, Alexander Striebeck, Mohammed S. Motawia, William G. T. Willats, Monica M. Palcic

**Affiliations:** ^1^Carlsberg Research LaboratoryCopenhagen, Denmark; ^2^Department of Plant and Environmental Sciences, University of CopenhagenFrederiksberg, Copenhagen, Denmark

**Keywords:** barley, starch synthases, biochemical characterization, substrate specificity, kinetics, affinity, stability, expression levels

## Abstract

Starch is the main storage polysaccharide in cereals and the major source of calories in the human diet. It is synthesized by a panel of enzymes including five classes of starch synthases (SSs). While the overall starch synthase (SS) reaction is known, the functional differences between the five SS classes are poorly understood. Much of our knowledge comes from analyzing mutant plants with altered SS activities, but the resulting data are often difficult to interpret as a result of pleitropic effects, competition between enzymes, overlaps in enzyme activity and disruption of multi-enzyme complexes. Here we provide a detailed biochemical study of the activity of all five classes of SSs in barley endosperm. Each enzyme was produced recombinantly in *E. coli* and the properties and modes of action *in vitro* were studied in isolation from other SSs and other substrate modifying activities. Our results define the mode of action of each SS class in unprecedented detail; we analyze their substrate selection, temperature dependence and stability, substrate affinity and temporal abundance during barley development. Our results are at variance with some generally accepted ideas about starch biosynthesis and might lead to the reinterpretation of results obtained *in planta*. In particular, they indicate that granule bound SS is capable of processive action even in the absence of a starch matrix, that SSI has no elongation limit, and that SSIV, believed to be critical for the initiation of starch granules, has maltoligosaccharides and not polysaccharides as its preferred substrates.

## Introduction

Storage of energy in the form of biopolymers is widespread in nature. Plants and green algae accumulate starch as their energy reserve. Starch is the main calorie contributor to the human diet, both directly through consumption of vegetable products and indirectly when used as animal fodder (Zeeman et al., 2010). World-wide starch production is currently larger than 3000 million metric tons (F.A.O., 2012) and it is estimated that it will have to double by 2050 to meet the nutritional needs of an increasing world population (Tilman et al., 2011). Starch is also used as an industrial raw product. While there is general knowledge of how plants synthesize starch, large gaps remain (Sonnewald and Kossmann, 2013) and details of how the amount of starch produced is regulated, and how enzymatic activity affects the internal structure and properties of starch are only known at a very basic level.

Barley is the fourth most abundant cereal crop. Traditionally, barley has been used for malting and brewing or as animal feed. However, it is gaining interest as a component of cereal-based foods for direct human consumption (Regina et al., 2012). Starch, accounting for up to 64% of the dry kernel weight in barley, can also be used for bioethanol production (Smith, 2008). Starch biosynthesis in the cereal endosperm (Emes et al., 2003; Zeeman et al., 2007; Blennow et al., 2013) is carried out by a set of enzymes including ADP-glucose pyrophosphorylase, starch debranching enzymes, branching enzymes, and starch synthase (SS) enzymes.

Starch is composed of two glucose polymers: amylose and amylopectin (Hassid, 1969). Both polymers consist of repeating glucose units that are α-1,4 linked. However, while amylose is predominantly linear, amylopectin contains branch points consisting of α-1,6-bonds. The branching pattern of amylopectin allows for the formation of the secondary and higher-order glucan structures that make up the matrix of every starch granule.

The exact molecular architecture of the starch granule is still unknown. It is thought that the combination of chain lengths, branching frequency and branching pattern in amylopectin give rise to a treelike structure in which clusters of glucose chains occur at regular intervals along the axis of an amylopectin molecule, with adjacent glucose chains in these clusters forming double helices (Pérez and Bertoft, 2010). They pack together in organized arrays giving rise to concentrically-arranged, crystalline lamellae in the granule matrix.

Starch synthases (SSs when in plural) are responsible for the growth of amylopectin and amylose molecules, with the soluble starch synthases SSI, SSII, SSIII, SSIV elongating amylopectin and granule bound SS (from now on GBSS) elongating amylose. They catalyze the addition of glucose from ADP-glucose (ADP-Glc) to the elongating chains of amylose and amylopectin, which then can become the substrates for other enzymes, most notably branching enzymes. The division of roles between the soluble SSs is often described as SSI, SSII, and SSIII elongating short, medium and long chains of amylopectin respectively, with SSIII and SSIV being involved in initiation through currently unknown mechanisms (Zeeman et al., 2010), but the exact division of roles in not known in detail.

Most studies to date have focused on the analysis of starch produced by mutant plants lacking or under-expressing one or more SSs. This can confound results as plastids contain different amounts of several SSs, which all catalyze the same reaction and compete for substrates. Furthermore, the relative abundances of enzymes vary with time, tissue and plant state, some enzymes may be posttranslationally modified and many can be found as part of complexes with other enzymes (Tetlow et al., 2008). Pleiotropic effects altering the amount of other SSs are also common in knockout mutants. Studies that biochemically analyzed semi-purified (Denyer et al., 1999b; Imparl-Radosevich et al., 1999b; Cao et al., 2000) or recombinantly produced (Imparl-Radosevich et al., 1998; Edwards et al., 1999; Commuri and Keeling, 2001; Bustos et al., 2004; Senoura et al., 2004, 2007; Busi et al., 2008; Valdez et al., 2008; Cuesta-Seijo et al., 2013) SSs have often focused on a few properties at a time, with methodology changing from study to study, which makes direct comparisons difficult.

In this study we have carried out the first extensive comparative biochemical characterization of all endosperm SS enzymes from barley. We analyzed and compared their kinetic constants, as well as their substrate specificities with numerous sugars including glucose, linear or branched oligosaccharides and polysaccharides. Furthermore, we analyzed their substrate affinity, processivity, and thermostability. In the case of SSI, the *in vitro* product profile and extension limit was studied.

There is currently no detailed information on the abundance of SSs during endosperm development in any crop plant. Previous studies have focused either on the detection of starch active proteins at a single time point in development, typically for only one protein; or on transcript levels throughout the development of the endosperm (Radchuk et al., 2009; Stamova et al., 2009; Kang et al., 2013). Transcripts, however, do not necessarily mirror the amount of active enzyme in the tissue (Gygi et al., 1999; Vogel and Marcotte, 2012). Here we present a temporal analysis of the abundance of SS enzymes during barley endosperm development, which will help to shed light on the involvement of each SS in starch biosynthesis in storage organs like endosperm.

A biochemical characterization of a complete enzyme class provides a framework for determining the contributions of the respective enzymes to starch production and structure and for comparison with SSs of other origins. Thus, we have created an experimental dataset from which the effects of each barley endosperm SS can be analyzed either in isolation or in comparison to those of all other SSs.

The activity assays used in this study detect directly either the activity or the products of the reaction, without subsequent steps involving modification or purification of the products that can lead to artifactual results. All assays were made with recombinant proteins free of contaminants with similar activities or likely to modify the products, and thus the properties measured can be attributed to the individual enzymes without fear of contaminating effects. This study is not meant to substitute, but rather to complement *in planta* studies, by providing a collection of experimental data points that go beyond what is possible to achieve *in planta*.

## Materials and methods

### Preparation of ADP-Glc

ADP-Glc diammonium salt was prepared using chemo-enzymatic synthesis as described in Cuesta-Seijo et al. (2013).

### Cloning of barley SSs

The gene of barley SSI (*Hv*SSI, Genbank accession: AAF37876.1) was codon optimized for *E. coli* expression using the online software tool GENEius (www.geneius.de) and synthesized by GenScript (www.genscript.com) in vector pUC57. Barley Granule bound SSI (*Hv*GBSSI, Genbank accession: AAM74048, in vector pJExpress414), starch synthases IIa (*Hv*SSIIa, Genbank accession: AAN28309, in vector pJExpress 411), IIIa (*Hv*SSIIIa, Genbank accession: AEL97583, in vector pJExpress 411), IIIb (*Hv*SSIIIb, Genbank accession: AFI61839, in vector pJExpress411), IV (*Hv*SSIV, Genbank accession: BAJ86666, in vector pJExpress 411) and the third CBM53 domain of *Hv*SSIIIa (in vector pET151) were codon optimized for *E. coli* expression and synthesized by DNA2.0 (www.DNA20.com). All genes were synthesized without their chloroplast transit peptide predicted using TargetP (Emanuelsson et al., 2000). The sequences used, including affinity tags, are listed in the Supplementary Information (S.I.). His-tags were placed in N-terminal positions for *Hv*GBSSI, *Hv*SSI, *Hv*SSIIa, and *Hv*SSIIIb, while they are in C-terminal positions for *Hv*SSIIIa, the third CBM53 domain of *Hv*SSIIIa and *Hv*SSIV.

### Expression and purification of barley SSs

Proteins were expressed in *E. coli* and purified as described previously (Cuesta-Seijo et al., 2013) with some alterations. Details are given in the S.I.

### Coupled spectrophotometric glycosyltransferase assay

Initial rates were determined by coupling the release of ADP to NADH oxidation via pyruvate kinase and lactate dehydrogenase in a protocol adapted from Gosselin et al. (1994). Assays were performed in a final volume of 100 μL with the following final concentrations: 50 mM Bicine, pH 8.5, 25 mM KOAc, 0.1% (w/v) bovine serum albumin, 2 mM MgCl_2_, 10 mM DTT, 0.375 mM NADH, 0.7 mM phosphoenolpyruvate tricyclohexylammonium salt, 6 U/mL pyruvate kinase, and 30 U/ml lactate dehydrogenase (both Sigma, rabbit muscle - type II) with 25–3800 nM enzyme at 37°C. Enzyme concentrations for activity assays were estimated by the method of Bradford with bovine gamma immunoglobulin as a reference and specific activities were normalized accordingly. 10 mM maltooligosaccharides (MOS), linear or branched, or 1 mg/ml polysaccharides were used as acceptors unless stated otherwise. Reactions were initiated by addition of 1 mM ADP-Glc. The reagents used as acceptors are listed in the S.I. NADH oxidation was monitored by the decrease in absorbance at 340 nm. For the soluble starches and amylopectins (maize and potato), stock solutions were heated to 90°C, vortexed and allowed to cool to room temperature shortly before use.

### Synthesis of AB-labeled maltohexaose

Maltohexaose (25 mg, 25 μmol) was reacted with 2-aminobenzamide (344 mg, 2.5 mmol) and NaBH_3_CN (160 mg, 2.5 mmol) at 60°C for 3 h in a mixture of DMSO (0.7 mL) and glacial acetic acid (0.3 mL). The reaction mixture was then diluted with H_2_O (10 mL), washed with dichloromethane (2 × 20 mL), concentrated *in vacuo* and then precipitated with EtOH. The product was purified by reverse-phase chromatography (Waters Sep-pak C18 plus cartridge, 0–50% (v/v) MeOH aq.).

### UPLC MOS length analysis

The labeled MOS were analyzed using UPLC (Waters Acquity UPLC, Waters BEH Glycan 1.7 μm (2.1 × 150 mm) column), equilibrated with 22% buffer A (10 mM ammonium formate, pH 4.5) and 78% buffer B (100% acetonitrile) and monitoring fluorescence (excitation 330 nm, emission 420 nm). Elongation reactions using the shorter version of *Hv*SSI were made in a buffer consisting of 200 mM NaCl, 8.3% (v/v) glycerol, 100 mM Tris pH 8.0 and 1 mM DTT. Fluorescent AB labeled maltohexaose was added to 100 μM. Varying levels of ADP-Glc were used. The protein was at 8 mg/mL final concentration and the reaction was incubated for 40 h (90 h in the reaction with 2.9 mM ADP-Glc) at 30°C or for the indicated amounts of time. The reactions (10 μL aliquots) were quenched with 50 μL of DMSO followed by 160 μL of a mixture of 78% acetonitrile and 22% (v/v) aqueous 10 mM ammonium formate pH 4.5, at which point they were used directly for injection (5 μL) into the UPLC machine. The AB labeled MOS were eluted using initially 22% of buffer A and 78% of buffer B (0.2 mL/min) followed by two consecutive linear gradients (22–50% of buffer A for 25 min at 0.2 mL/min and from 50 to 70% buffer A for 20 min at 0.1 mL/min). The elongation and reverse reactions using wild type *Hv*SSI were carried out in the same buffer but with 2 mg/mL protein and 30 μM AB labeled maltohexaose. The reactions (10 μL aliquots) were quenched with 90 μL of DMSO then a 10 μL aliquot was diluted with 190 μL of a mixture of 78% acetonitrile and 22% (v/v) aqueous 10 mM ammonium formate pH 4.5 and used directly for injection (5 μL). The AB labeled MOS were eluted using initially 22% of buffer A and 78% of buffer B (0.2 mL/min) followed by two consecutive linear gradients (22–50% of buffer A for 25 min at 0.2 mL/min and from 50 to 100% buffer A for 5 min at 0.1 mL/min).

### Estimation of reaction rates based on product size distribution

For the estimation of the decay of reaction velocity with DP of the MOS acceptor, an ad hoc simulator was built in Excel. Details of the procedure are given in the S.I.

### Determination of the mode of action of SSs

For SSI, SSIIIb, and SSIV 100 μL of 50 mM Bicine, pH 8.5, 25 mM KOAc, 1 mg/mL BSA, 5 mM MgCl_2_, 10 mM DTT, 5 mM maltotriose, and 0.1 mg/mL enzyme were mixed and the reaction was started by the addition of 7.5 mM ADP-Glc. The reaction was incubated at 30°C for 24 h and stopped by boiling the sample for 5 min at 95°C. The reactions with SSIIa and GBSSI were made in 250 μL of 50 mM Bicine, pH 8.5, 25 mM KOAc, 10 % (v/v) glycerol, 2 mM MgCl_2_, 2 mM DTT, 5 mM maltotriose or maltooctaose, and 0.3 mg/mL SSIIa or 1 mg/mL GBSSI. An aliquot of 20 μL was removed and served as control before the addition of 7.5 mM ADP-Glc. Further aliquots were removed at 10, 30, 60, 120, 360, 720, and 1440 min, immediately boiled for 2 min at 95°C and kept frozen until further use. Samples were labeled essentially as described in *Synthesis of AB-labeled maltohexaose*, except that for the 20 μL aliquots only 100 μL of 1 M 2-aminobenzamide and 100 μL of 1 M NaBH_3_CN solution in DMSO:acetic acid (7:3), 2 mL of water, and 2 × 5 mL dichloromethane were used. Samples were diluted 1:10 in 78% acetonitrile and 22% (v/v) aqueous 10 mM ammonium formate pH 4.5 and analyzed on a UPLC machine as described above.

### NMR

The product formation from branched substrates (isomaltose and glucosyl-maltotriose) was confirmed by monitoring the reaction using NMR spectroscopy. All NMR spectra were recorded at 37°C on a Bruker Advance DRX 800 instrument. Acceptor substrate (10 mM final concentration) and ADP-Glc (5 mM final concentration) were weighted out, dissolved in 100 mM deuterated phosphate buffer (pH 7.5), 1 mM DTT, and transferred to a 5 mm NMR-tube. The substrate mixtures were initially analyzed for impurities before adding 20 μL concentrated proteins.

### Differential scanning fluorimetry

*Hv*SSI unfolding temperatures were measured with a variant of the Thermofluor method (Niesen et al., 2007). Details are given in S.I.

### Glycan microarrays: oligosaccharide samples

Oligosaccharides were either purchased from Sigma-Aldrich Co. or Megazyme International, or prepared by chemical synthesis. Branched and phosphorylated maltooligosaccharides were synthesized (Motawia et al., 1995, 2005; Sakairi et al., 1995; Damager et al., 2005; Hansen et al., 2009). Native starch granule samples from potato, maize, waxy maize, pea, tapioca, and wheat were obtained from KMC (Brande, Denmark). Amylopectin isolated from maize and potato and amylose isolated from potato tuber were from Sigma-Aldrich Co. The different molecular structures of these starches were described elsewhere (Blennow et al., 2000). Carbohydrate microarrays where printed as described on nitrocellulose membranes (Pedersen et al., 2012) with spots formed by 600 μL with concentrations of 2 and 10 mg/mL for polisaccharides and MOS respectively. All starches were solubilized in NaOH prior to printing (Pedersen et al., 2012). *Microarray Probing*–microarrays where probed as described (Pedersen et al., 2012). Briefly, the arrays where probed with a set of His-tagged SS proteins, His-tagged carbohydrate binding modules (CBMs) and monoclonal antibodies (PlantProbes, Leeds, UK). SS proteins and CBMs were diluted in PBS containing 5% (w/v) low fat milk powder (MPBS) to 30 μg/mL protein, and antibodies to 1/10 respectively. For detection, secondary anti-His or anti-rat antibodies conjugated to alkaline phosphatase (Sigma) were diluted in MPBS to 1/5000. Developed arrays were scanned at 2400 dpi (CanoScan 8800F), converted to TIFFs and signals were measured using Array-Pro Analyzer software (Version 6.3, Media Cybernetics). Data are presented in a heatmap, where color intensity is correlated to mean spot signals. A cut off of 5 units was applied. Cloning, expression and purification of the CBM20 from *Aspergillus niger* (Christiansen et al., 2009) is described in the S.I.

### Plant material and tissue preparation

Barley plants (*Hordeum vulgare* “QUENCH”) were cultivated under standard greenhouse conditions at 18°C with 16 h of light and a relative air humidity of 60%. Developing seeds were harvested from the middle region of the ear at 2 day intervals starting from anthesis until 24 days after flowering (DAF). Pericarp and endosperm tissue fractions were separated by hand dissection. The plant material was immediately transferred onto dry ice. Using a mortar and pestle, the frozen material was powdered on dry ice. Subsequently the powder was dissolved in extraction buffer: 100 mM MOPS pH 7.5, 150 mM NaCl, 0.1% (v/v) Triton X-100, 10% (v/v) glycerol, 1 mM DTT, 5 mM EDTA, 1% (w/v) polyvinylpyrrolidone, plant protease inhibitor (P9599–Sigma Aldrich), and processed with a glass homogenizer placed on ice. The resulting plant protein extract was separated into buffer soluble and buffer insoluble protein extract by centrifugation at 22000 g for 30 min at 4°C.

### Immunoblot analysis

Purified recombinant *Hv*GBSSI, *Hv*SSI, *Hv*SSIIa, *Hv*SSIV, *Hv*BEI, and *Hv*BeIIb were used to immunize rabbits (Genscript®, USA Inc. 860 Centennial Ave. Piscataway, NJ 08854 USA). Bleeds were taken every seven days during the course of 4 weeks. The serum of the final bleed was used in immunological western blot experiments. Primary antibodies were used at a final concentration of 1:250. Secondary antibodies (Cy5-conjugated anti rabbit monoclonal antibodies) were used at a final concentration of 1:1500. To obtain semi quantitative data from the immunoblots the freely available ImageJ software (http://imagej.nih.gov/ij/) was used to quantify bands based on known protein standards.

## Results

### Substrate specificity of all endosperm SSs

The activity of each purified SS with a series of potential acceptor substrates was evaluated with a coupled spectrophotometric assay at 37°C unless otherwise noted. As elaborated in the discussion, it is not completely clear which (or whether both) of the SSIII isoforms is expressed in endosperm. Consequently, some of the experiments were done with both isoforms to ensure completeness.

#### Activity profile of all SSs with maltooligosaccharides (MOS) as acceptors

**Figure 1:**
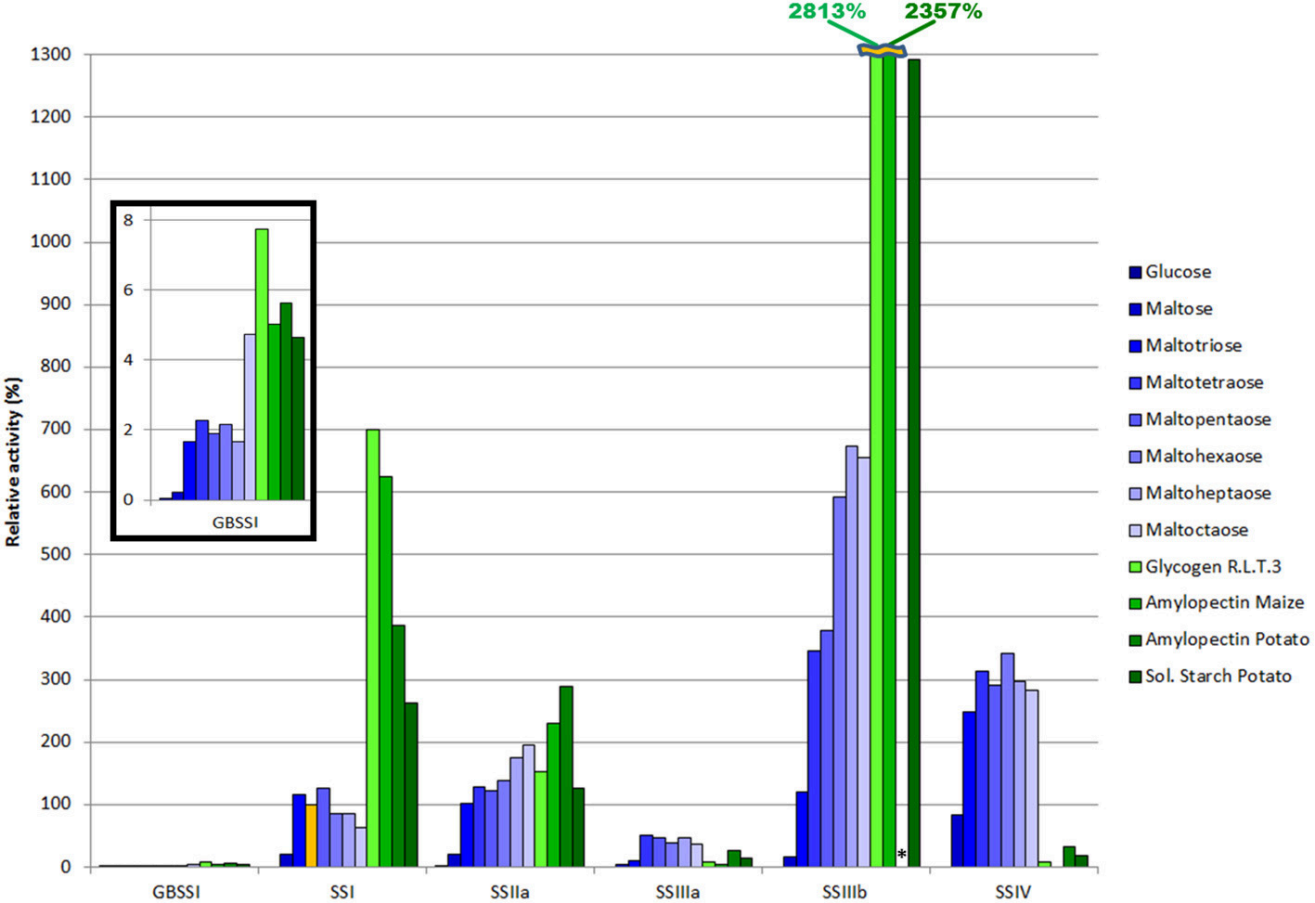
**Substrate specificity of SSs**. Relative activity for all SS enzymes with different MOS and polysaccharides as acceptors, assayed at 37°C with 1 mM ADP-Glc, 10 mM acceptor for MOS (eight blue bars, from darker for DP = 1 to lighter for DP = 8) and 1 mg/mL acceptor for branched polysaccharides (four green bars). All specific activities are normalized to the values with maltotetraose for SSI (orange bar), which corresponds to 0.302 μmol·min^−1^·mg^−1^. Two values that would require the y-axis scale to be doubled are shown numerically with broken bars. The asterisk denotes a value which was not determined.

All MOS from DP1 (glucose) to DP8 (maltooctaose) were tested as acceptors for the SSs. These results, along with the results for polysaccharides, are shown in Figure 1. MOS were limited to DP8 since this is the longest commercially available compound. In the case of SSI we extended the experimental data beyond DP8 (see below). No significant activity was detected with glucose as acceptor substrate. However, a small activity could be measured at very high concentrations (1 M) of glucose, which is compatible with the presence of a maltose impurity in our glucose stock (up to 0.2% according to the manufacturer).

Maltose acted as an acceptor substrate for all SSs tested with clearly measureable activity, although at a level several fold lower than that with longer MOS. *Hv*GBSSI had an almost constant activity profile from DP3 to DP7 with a sharp 2.8 fold increase in activity from DP7 to DP8. For *Hv*SSI there is a slight increase in activity from DP3 to the maximum at DP5 followed by a decline in activity for longer DPs, with DP8 showing 50.2% of the activity with DP5. *Hv*SSIIa showed a consistent increase in activity with increasing acceptor lengths, and if it has an activity maximum it will likely be for chains with DP > 8. For SSIIIa, SSIIIb and SSIV, DP3 as an acceptor results in a reaction rate 3–6 times lower than for DP4-DP8. The reaction rate profile for DP4-DP8 was approximately flat for *Hv*SSIIIa, but activity increased with acceptor length for *Hv*SSIIIb. The activity of *Hv*SSIV was maximal when tested with DP6 as the acceptor, with minor declines for longer and shorter substrates between DP3 and DP8.

#### Starch and glycogen are good acceptors for all SSs except SSIV

We measured the activity of each enzyme with a series of branched polysaccharides as acceptors, which more closely resemble the expected natural substrates of SS activity (Figure 1, green bars). When compared to maltotetraose, the shorter MOS recognized as a good substrate by all enzymes, the activity level on branched oligosaccharides was 2–3 fold higher for *Hv*GBSSI, 3–7 fold higher for *Hv*SSI, comparable to two times higher for *Hv*SSIIa and 4–8 times higher for *Hv*SSIIIb. In the case of *Hv*SSIIIa the branched polysaccharides were 2–12 fold slower acceptors, and in the case of *Hv*SSIV, branched polysaccharides were so slow acceptors that the activity was undetectable with maize amylopectin. *Hv*GBSSI, *Hv*SSI, and *Hv*SSIIIb preferred the short chain substrate glycogen, while for *Hv*SSIIa the activity was higher with the longer chain substrates. *Hv*SSIIIa and *Hv*SSIV displayed mixed results in this respect.

#### Other acceptor substrates

We also tested the activity of all SSs except *Hv*SSIIIb with a series of small branched and modified linear acceptors, see Figure S1. Measured activities were small in all cases except for maltosyl trehalose, which features a maltotriose unit at the non-reducing end. Maltosyl-β-cyclodextrin and maltosyl-maltotriose were also recognized and elongated at a significant rate, in the order of that for maltose. All other substrates displayed slower elongation rates.

#### UDP-glucose is a substrate for GBSSI, SSI, and SSII but not for SSIII, SSIV

Some SS enzymes can utilize UDP-Glc as a donor instead of ADP-Glc. We tested *Hv*GBSSI, *Hv*SSI, and *Hv*SSIIa with 100 mM UDP-Glc and 1 mg/mL glycogen as acceptor. They showed activities of 2.3, 4.1, and 2.3% respectively compared to those measured with 1 mM ADP-Glc as acceptor. *Hv*SSIIIa and SSIV showed no measureable activity with 100 mM UDP-Glc and 10 mM maltotriose, while *Hv*SSIIIb was not assayed with UDP-Glc.

#### SSIIIb has the highest specific activity amongst all SSs

Selecting the activity of *Hv*SSI with DP4 as the reference for all activities provides an impression of the relative specific activities of each enzyme (Figure 1). *Hv*SSIIIb displayed the highest specific activity both with linear and branched sugars as acceptors, being at least 4 times higher than any other SS for most substrates with the exception of SSIV with linear MOS. SSIV, with MOS as substrate, has a specific activity about twice that of *Hv*SSIIa, 3 times that of SSI and 7 times faster than *Hv*SSIIIa. For polysaccharides, SSI showed an activity about twice that of *Hv*SSIIa, although in a chain length dependent manner, while *Hv*SSIIIa and *Hv*SSIV had activities low enough to be insignificant in comparison. *Hv*GBSSI displayed an activity much lower than any other SS with all substrates.

### Kinetic constants of all SS enzyme classes

We used our *in vitro* coupled spectrophotometric metric assay for Michaelis-Menten kinetics for several enzyme-substrate combinations, which are given in Table 1 and Figure S4. K_cat_ values were measured but are in most cases just a lower limit estimation as full saturation (defined as at least 10 times the K_M_ concentration) was in general not achieved simultaneously for donor and acceptor. The reported K_M_ values do not suffer from this problem. Measured K_cat_ values follow the general trend already reflected in Figure 1, with *Hv*SSIIIb displaying the highest activity levels in the order of 1000 turnovers per minute followed by *Hv*SSIV (2-fold lower but measured only with MOS). Several-fold lower K_cat_ values were obtained for *Hv*SSI and *Hv*SSIIb, in the range of one to two reaction cycles per second; and again several-fold lower values for *Hv*GBSSI.

**Table 1:** **K_M_ and K_cat_ constants for all SS classes**.

**Protein**	**K_M_ (ADP-Glc)**	**K_M_ (glycogen)**	**K_M_ (MOS)**	**K_cat_ (turnovers·min^−1^)**
GBSSI	0.09 ± 0.02 mM (DP = 2)	0.077 ± 0.008 mg/mL^*^	46.32 ± 1.05 mM (DP = 5)^*^	13.5 ± 0.2 (DP = 5)^*^
	0.18 ± 0.02 mg/mL (glyc.)			13.4 ± 0.5 (glyc.)^*^
	0.09 ± 0.02 gm/mL (amy.)			
SSI	0.63 ± 0.02 mM (DP = 3)	N.A.^**^	N.A.	>>70 (DP = 5)^**^
	0.60 ± 0.12 mg/mL (glyc.)			>70 (glyc.)^**^
SSIIa	0.22 ± 0.02 mg/mL (glyc.)	0.49 ± 0.03 mg/mL	N.A.	>>60 (DP = 5)
				68.2±0.9 (glyc.)
SSIIIb	0.44 ± 0.04 mM (DP = 3)	1.2 ± 0.2 mg/mL	N.A.	>740 (DP = 3)
				1073 ± 39 (glyc.)
SSIV	0.31 ± 0.02 mM (DP = 3)	N.A.	20.41 ± 1.00 mM (DP = 5)	533 ± 10 (DP = 5)

In brackets, the type of acceptor used except in the first row, where it the variable (“glyc” = glycogen, “amy” = amylopectin). Glycogen is at 0.1 mg/mL except in the second and fourth columns, DP2 at 100 mM, DP3, and DP5 at 10 mM except in the fourth column where they were the variable. N.A. = Not available (not conforming to Michaelis Menten under attainable experimental conditions in the cases of SSI, SSII, and SSIII, too low activity in the case of SSIV).

* Measured at 30°C.

** Data from Cuesta-Seijo et al. (2013).

Values for K_M_s for ADP-Glc were measured with constant acceptor concentrations (Figure S4A). In some cases it was impossible to achieve acceptor saturation (see below). Of all five enzymes tested, *Hv*GBSSI has the highest affinity for the ADP-Glc donor with measured K_M_ in the order of 0.1 mM depending on the acceptor. In contrast *Hv*SSI, has the highest K_M_ values for ADP-Glc, at approx. 0.6 mM, a value three times higher than for *Hv*SSIIa. Donor affinities measured for *Hv*SSIIIb and SSIV are 0.44 and 0.31 mM, respectively. In the cases where K_M_ for ADP-Glc was measured both with linear and branched substrates the results were similar. *Hv*SSIIa measurements were only obtained with glycogen, while for *Hv*SSIIIb and *Hv*SSIV only in the presence of linear MOS. It is reasonable to expect similar values for the alternative substrate in these cases as well.

Measurements of K_M_ for acceptors were carried out using 1 mM ADP-Glc (Figure S4B). While for most enzymes this value is below saturation, it is in all cases well above K_M_ for the donor. We obtained K_M_ values for glycogen of 0.0774 mg/mL for *Hv*GBSSI, 0.489 mg/mL for *Hv*SSIIa and 1.21 mg/mL for *Hv*SSIIIb. For *Hv*SSIV the activity with glycogen was too low to determine a K_M_. It was already reported (Cuesta-Seijo et al., 2013) that *Hv*SSI could not be saturated with glycogen or soluble starch under the attainable experimental conditions.

We also collected data on the variation of SS activity with the concentration of linear MOS as acceptors (Figure S4C). K_M_ values of 46.32 mM and 20.41 mM were determined for GBSSI and SSIV respectively with maltopentaose as the acceptor. These values correspond to about 4 and 2% (w/v) acceptor concentrations. With *Hv*SSI, *Hv*SSIIa, and *Hv*SSIIIb only minor signs of saturation were observed at concentrations as high as 160 mM DP3 with *Hv*SSIIIb, 100 mM DP5 with SSI and 125 mM DP5 with *Hv*SSIIa. If these enzymes can be saturated with MOS, it will only be at much higher, non-physiological concentrations.

#### The elongation reaction of SSI is a reversible equilibrium

Starch synthesis is normally considered to be a unidirectional reaction. Brust et al. (2013) showed that *At*SSI can, in the presence of excess ADP, catalyze the reverse reaction with shortening of the glucose chain and production to ADP-Glc. We tested whether *Hv*SSI showed the same behavior using a fluorescently labeled maltohexaose as the starting material with UPLC analysis of MOS.

**Figure 2:**
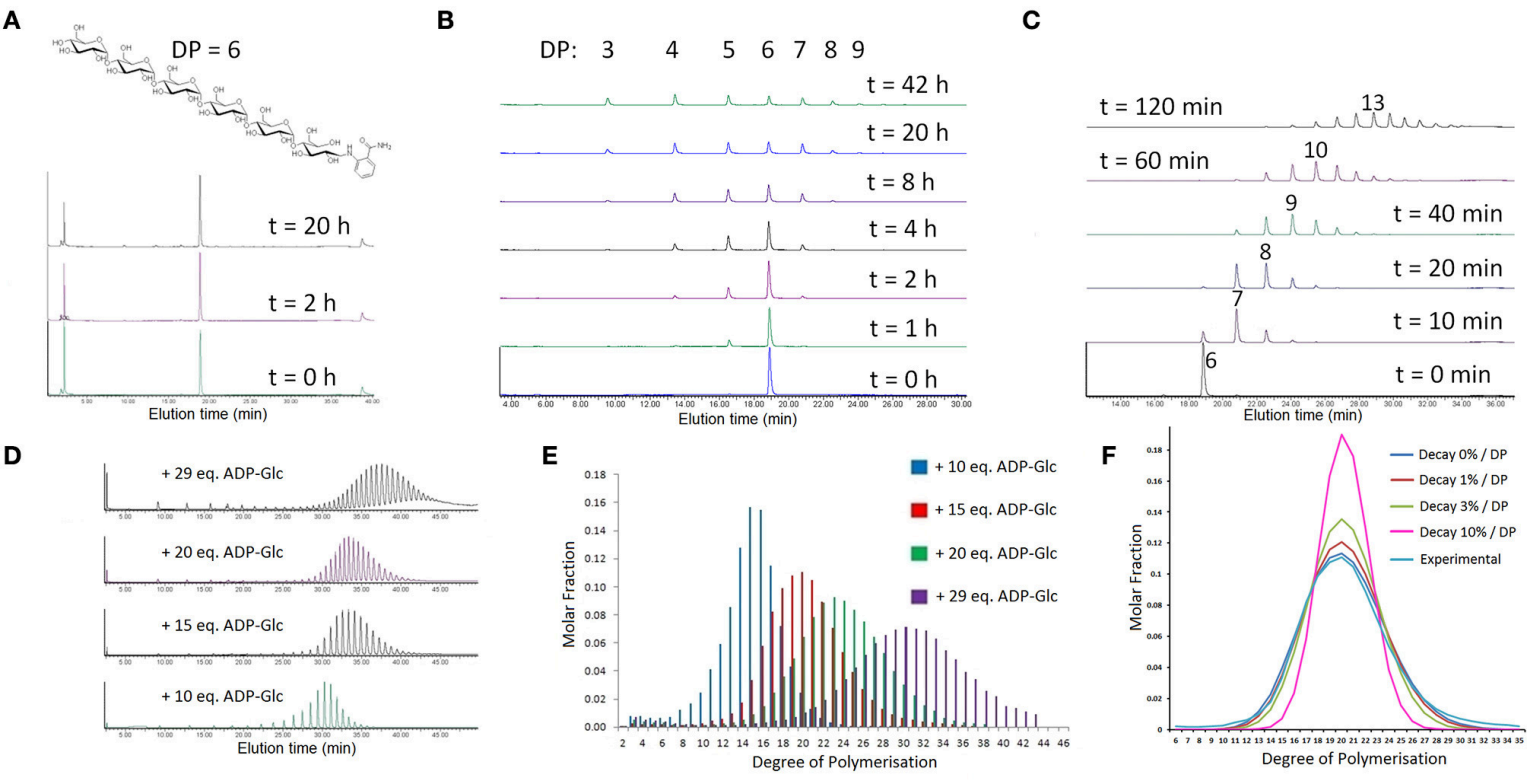
**Reversibility and long-range elongation profile of SSI**. **(A)** Structure of the fluorescently labeled acceptor and proof of the absence of any reaction in the absence of both ADP and ADP-Glc, but in the presence of enzyme and fluorescent acceptor. **(B)** UPLC reaction profile over time after addition of 10 mM ADP. **(C)** UPLC reaction profile over time after addition of 5 mM ADP-Glc. **(D)** UPLC reaction profiles with a truncated version of *Hv*SSI after addition of variable amounts of ADP-Glc, measured after all donor was depleted. Only for panels **(D–F)** in this figure are the data originating from the truncated version of *Hv*SSI. **(E)** Molar fraction of each MOS species after integration of peak areas from panel **(D)**. **(F)** Models of expected MOS species distributions with different kinetic models, compared with the molar fractions from panel e (“Experimental” corresponds to the red bars in panel **E**).

In a control reaction where neither ADP-Glc nor ADP was added, the substrate remained unmodified (Figure 2A). When we used 10 mM ADP in the reaction, *Hv*SSI initially and very slowly shortened the substrate (Figure 2B). As shorter MOS accumulate, the forward elongation reaction starts to be evident, proving that the shortening reaction produced ADP-Glc and is thus the reverse of the elongation reaction and not hydrolysis. The reaction with 5 mM ADP-Glc resulted in elongation and a moving distribution of products (Figure 2C). Quantitation of peak areas showed the forward reaction with 5 mM ADP-Glc was 46.9-fold faster than the reverse reaction with 10 mM ADP. The difference in the rate for the forward and reverse reactions might well be much larger than this after accounting for enzyme saturation.

#### SSI is not sensitive to acceptor length and does not have an elongation limit

We tested how far *Hv*SSI would elongate MOS *in vitro* using the same fluorescently labeled substrate as above at 0.1 mM concentration. For these assays the enzyme was changed to a modified version of *Hv*SSI shortened by 84 amino acids at the N-terminus, the same enzyme referred to as “rice like” in Cuesta-Seijo et al. (2013). It has an intact catalytic domain and lacks the disordered N-terminal tail analogously to mature rice and maize SSI (Imparl-Radosevich et al., 1998), but it is several times faster than wild type *Hv*SSI. The use of this truncated version was necessary as reaction times were otherwise estimated to be several days. Rather than by a time series, the end points of the reactions were controlled by limiting the amount of ADP-Glc available, with the reactions stopped only by depletion of ADP-Glc. 1, 1.5, 2, and 2.9 mM ADP-Glc were used in four separate reactions limiting the extent of elongation to 10, 15, 20, and 29 reaction cycles on average, respectively. The UPLC traces are shown in Figure 2D and quantitation of the resulting peak areas in Figure 2E. The measured net number of forward reaction cycles was 8.8, 12.3, 17.5, and 23.6 respectively. The discrepancy could be explained by several factors: slow hydrolysis of ADP-Glc and a problem with solubility limiting the amount of MOS with DP > 20 actually being injected in the UPLC, which was only partly corrected by addition of DMSO; as well as limited baseline resolution for DP > 30. The result of the reactions is a distribution of products with Gaussian profiles, peaking at DP = 15, 20, 23, and 30 respectively. The shape and height of each product distribution simply reflects formation of a wider array of products as the reaction progresses, with no signs of the nature of the reaction changing as DP increases. In the reaction with the largest amount of ADP-Glc, single peaks can be distinguished and quantitated up to DP = 43, with the elevated baseline at longer elution times suggesting the presence of products with DP = 50 or longer.

To estimate the relative reaction rates for acceptors with DP > 8, which were not available for the spectrophotometric assay, we used the product distribution with 1.5 mM ADP-Glc (15 eq.), which appears to be free of precipitation. The details of the calculation are described in the S.I., and the results are shown in Figure 2F. The best fit to the experimental data is for a model where the reaction rate is constant for DP > 8, that is, for a scenario where all acceptors larger than DP 7 are equally available to *Hv*SSI. Models in which longer acceptors become progressively worse substrates result in narrower product distributions. The reverse reaction, not included in the model, might have taken place to a certain degree resulting in broadening of the experimental distribution, but it is safe to assume that, if there is a reduction in the reaction rate with increasing DP, it is very small. It can be inferred that no considerable increases in affinity or reductions in the reaction rate are present at least to DP = 25; and considering the shape of the other product distributions, at a qualitative level, to DP = 40. This method of fitting with this particular reaction stoichiometry, while good in the long range, would not discriminate if there was a reduction in reaction velocity between DP = 8 and approx. DP = 12 followed by a stabilization from there on. The forward, time-limited reaction series with wild type enzyme described before (Figure 2C) allows us to fill that gap. As shown in Figure S2, the behavior is approximately linear from a point where DP = 6 to a point with average DP = 13.5.

#### Soluble SSs behave distributively, GBSSI is distributive *in vitro* up to DP < 7 but changes behavior for DP > 7

We performed specific experiments to attempt to identify the product signature of a possible processive mechanism (where the same substrate molecule is used for further reaction cycles). We observed the same behavior as for SSI, that is, a distributive reaction mechanism with release of products after each reaction cycle, for the other soluble SSs tested: *Hv*SSIIa, *Hv*SSIIIb, and *Hv*SSIV. Details on those enzymes, including further SSI elongation profiles with maltotriose as acceptor, are described in Figure S3.

**Figure 3:**
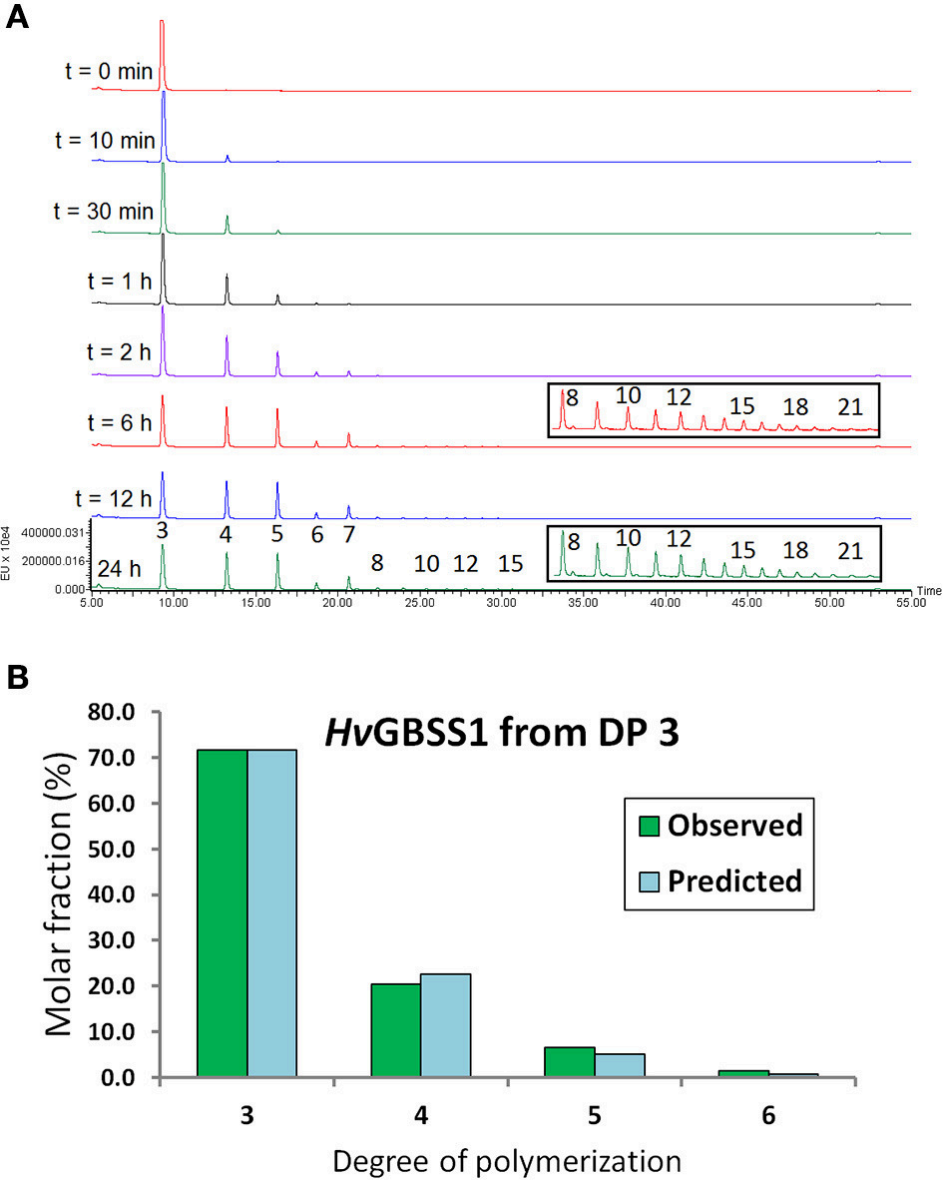
**Observed and predicted reaction course for *Hv*GBSSI action on maltotriose**. **(A)** UPLC traces of reaction of GBSSI with unlabeled maltotriose. DPs of product and time points are indicated. Labeling was done after reaction termination. The DP = 3 peak is truncated in all traces up to 2 h for improved visibility of the other peaks. Boxes contain the traces for the larger peaks with a zoomed in vertical scale to illustrate the relative areas of peaks up to DP = 21, some peaks with even larger DPs are visible. **(B)** Observed (green bars) and predicted (blue bars) product distributions corresponding to the reaction progress after 6 h, before DP = 7 started to accumulate. The predicted product distribution was calculated assuming a purely distributive reaction mechanism and the individual reaction velocities for each acceptor measured at 37°C.

We measured the reaction profile for *Hv*GBSSI starting from DP = 3 and DP = 8 as a time series with 1.5 equivalents of ADP-Glc. Starting from DP = 3 the behavior is analogous to that of the soluble SSs up to the point where DP = 7 starts to accumulate (Figure 3A), with the experimental and calculated product distributions matching up to that point (Figure 3B). The behavior then changes with the peak for DP = 6 becoming smaller and the peak for DP = 7 becoming larger, eventually doubling the area of the DP 6 peak (Figure 3A). The peak for DP 8 is then 8 fold smaller than that of DP = 7 and it is followed by a very long tail of peaks for DP > 8 which all appear more or less simultaneously and whose area decays only slowly with increasing DP (Figure 3A). None of these two behaviors could be explained with our distributive model of SS action. The reaction was almost completed after 6 h consistent with donor depletion and only small variations are observed from that time point onwards. In the time series starting with DP = 8 the reaction was essentially complete after 1 h with only minor elongation reactions occurring to the species of DP = 6 or 7 and essentially none to DP = 3, 4, or 5. In the same time period, the amount of DP = 8 diminishes dramatically substituted by peaks up to DP = 18 which appear without the amount of DP = 8 ever diminishing below that of DP = 9 (Figure S3D). Elongation after 1 h might predominantly be a consequence of ADP-Glc formation from the reverse reaction as evidenced by an increase in the amounts of the shorter MOS.

### Temperature stability of all SSs

**Figure 4:**
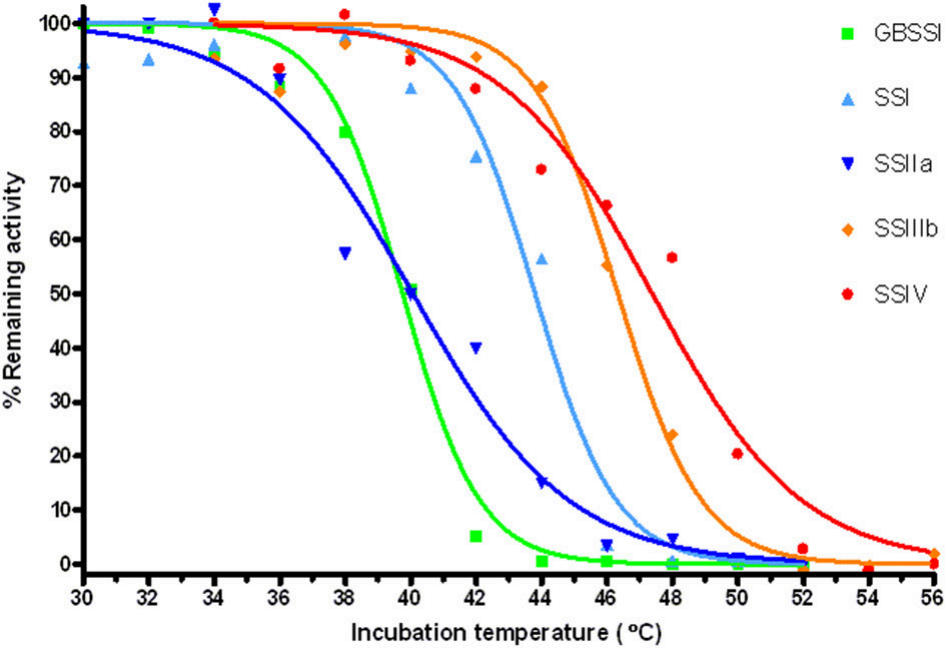
**Temperature stability of the SS enzymes**. Residual activity of SS samples after incubation at the indicated temperatures. Solid lines are sigmoidal fits to the experimental data.

We assayed the thermostability of the SS enzymes *in vitro*. The enzymes were incubated at different temperatures for 15 min and the remaining activity measured at 30°C with the spectrophotometric assay. Details are given in S.I. and results are shown in Figure 4. The temperatures at which only 50% of the activity remains after incubation are 39.8°C for GBSSI, 40.1°C for *Hv*SSIIa, 43.8°C for SSI, 46.3°C for *Hv*SSIIIb, and 47.4°C for *Hv*SSIV. A similar trend, with *Hv*GBSSI and *Hv*SSIIa displaying the lowest thermostability followed by SSI and with *Hv*SSIIIb and *Hv*SSIV showing the highest thermostability, was observed for the temperature ranges bracketing relative activities between 90 and 5% of controls. These temperatures are 36–42°C for *Hv*GBSSI, 36–46°C for *Hv*SSIIa, 40–46°C for *Hv*SSI, 44–50°C for *Hv*SSIIIb, and 42–52°C for *Hv*SSIV.

The effect of additives on SSI stability was studied in some detail with the thermofluor assay; details for many additives are given in S.I. The strongest effects were from sugars, which increased protein stability by 1°C for every 50 mM hexose present as oligosaccharides, with glucose and other monosaccharides having only half the effect. Glycerol also had a strong effect, stabilizing *Hv*SSI by almost 1°C for every 3% (v/v) present. On the contrary salts had a destabilizing effect, in the order of 1°C for every 0.3 M NaCl added. From this data it can be expected that the thermostability of all SSs will be higher in the sugar-rich plastid environment than what our activity assay suggests.

#### Temperature dependence of SS activity

**Figure 5:**
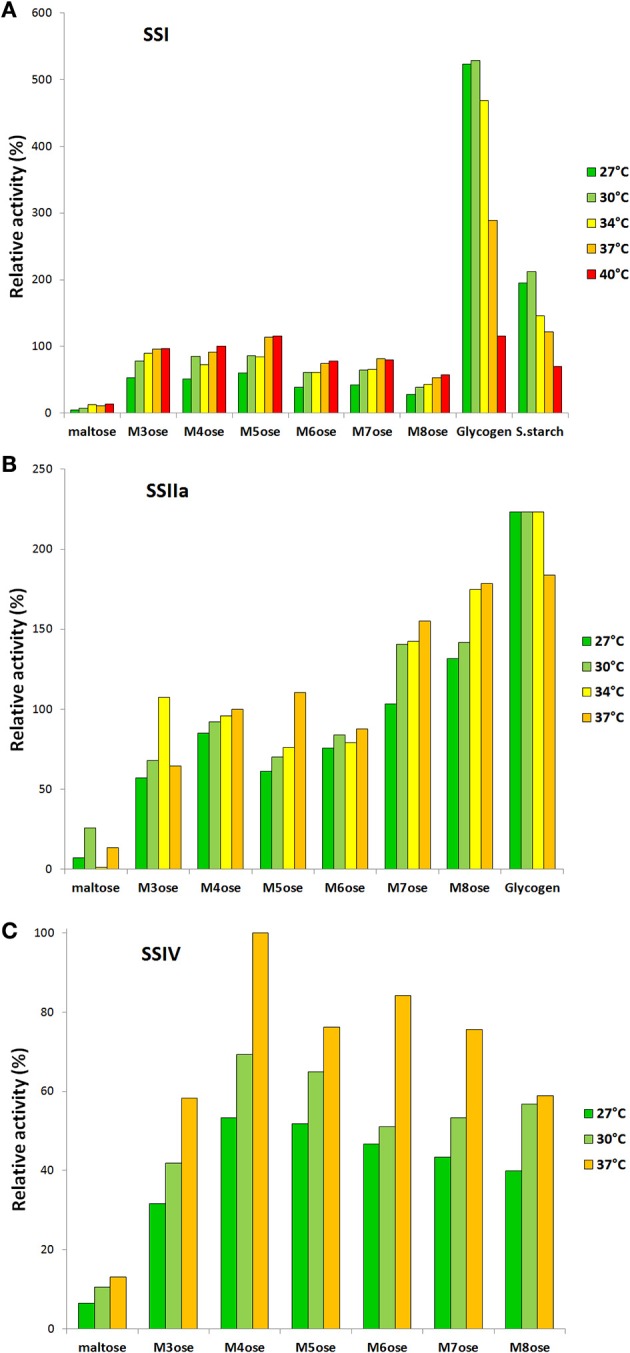
**Variation of SS activity with temperature**. **(A)** Compared activity of SSI with different acceptors at different temperatures. The first seven are linear MOS, the last two branched polysaccharides, assayed at 0.1 mg/mL. All data presented as relative values to maltotetroase at 37°C. **(B)** The same for SSIIa, glycogen at 1 mg/mL concentration. **(C)** The same for SSIV. The assay temperatures are different from panel to panel, as are the gaps between temperatures, but the color scheme has been kept consistent.

The temperature dependence of *Hv*SSI, *Hv*SSIIa, and *Hv*SSIV activity *in vitro* was measured with the coupled spectrophotometric assay (Figure 5). The enzymes were tested against different linear and branched substrates at 27, 30, 34, 37, and 40°C or subsets thereof. The behavior is the same for all three enzymes with all MOS tested as acceptors, with a gradual increase in activity with temperature in the order of 50% between 27 and 37°C. The maximum activity takes place at 40°, where only *Hv*SSI was assayed, but this is only slightly higher than at 37°C. Possibly, limited thermostability of the enzyme at 40°C starts to negatively affect the measured activity. The situation is different when glycogen is used as substrate: In the case of *Hv*SSI, the maximum activity is at 30°C, with activity at 27°C being 1.1% lower. The activity then drops at higher temperatures with an average reduction of activity of 46.3% between 30 and 37°C and of 78.1% to 40°C. The latest drop in activity cannot be attributed to protein stability since it is not present with MOS as substrates. SSI with soluble starch as acceptor showed a similar behavior with drops of activity of 42.5 and 66.1% respectively. The activity of *Hv*SSIIa was much less sensitive to temperature variations with glycogen as a substrate, staying constant between 27 and 34°C and experiencing a drop of only 17.6% at 37°C. *Hv*SSIV was not assayed for temperature sensitivity with any branched polysaccharides due to its low activity on such substrates.

### Microarray study of binding affinities of SSs in isolation

**Figure 6:**
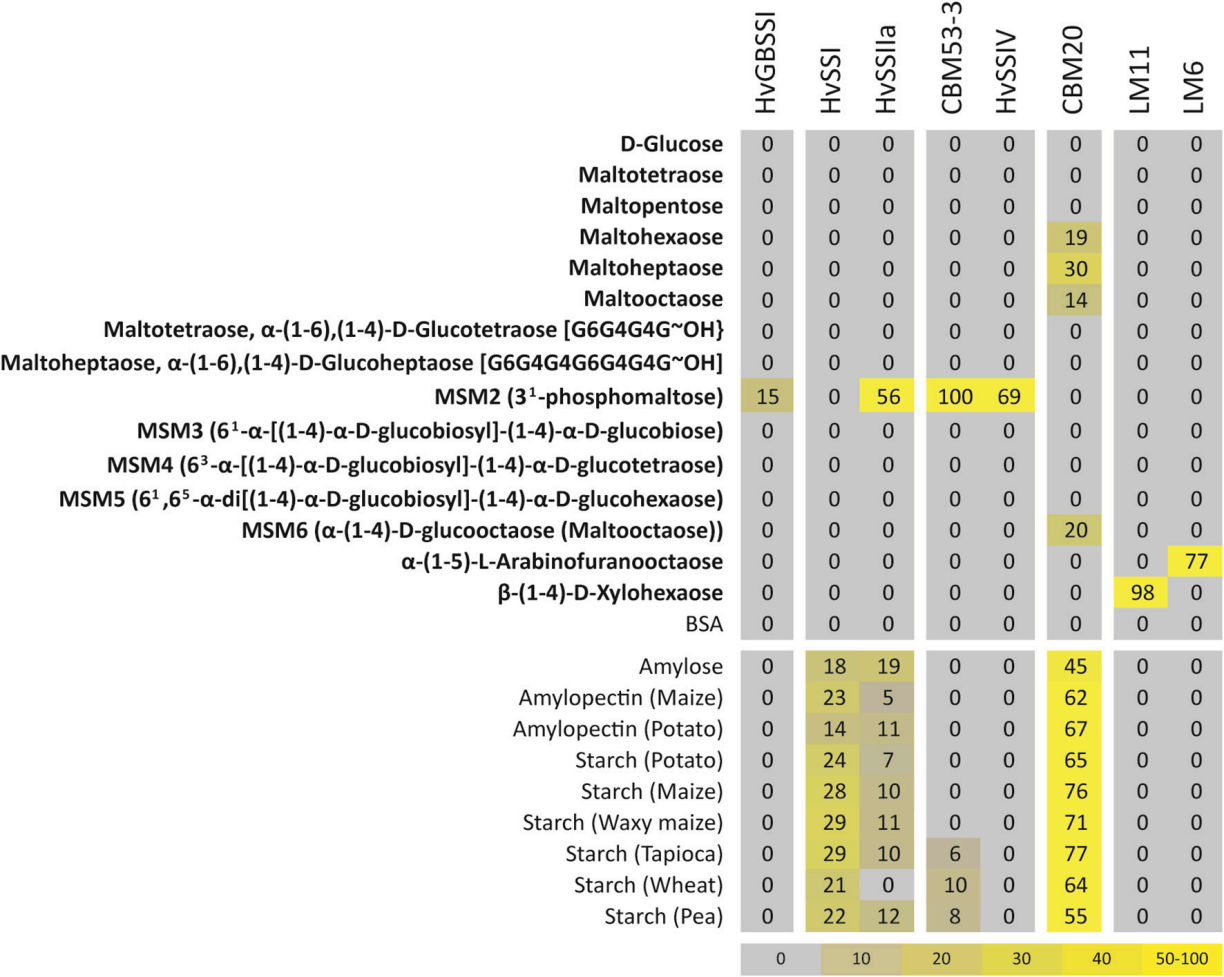
**Affinity of SSs for different substrates from glycan arrays**. Carbohydrate microarray binding profile, which shows binding of SSs to various poly- and oligosaccharides. In addition, positive controls CBM20 (binding to starch), LM11 (binding to (1,4)-β-D-xylan/arabinoxylan) and LM6 (binding to (1,5)-α-L-arabinan) were included. The mean spot signals obtained from four experiments are presented in a heat map in which color intensity is correlated to signal. The highest signal in each data set was set to 100, and all other values were normalized accordingly as indicated by the color scale bar.

All SSs are in some way associated with the starch granule. This can be brought about by direct interaction of the proteins with the starch granule or via protein-protein interaction. To test starch interaction *in vitro* we probed all SS with a carbohydrate array that was printed with a large variety of different starches and maltooligosaccharides (Figure 6). Samples of *Hv*SSIIIa and *Hv*SSIIIb did not show binding to any substrates, possibly an experimental artifact, and were left out of the final dataset. The third carbohydrate binding domain of *Hv*SSIIIa (CBM53-3) was included as a proxy for *Hv*SSIII.

*Hv*GBSSI and *Hv*SSIV did not show any binding to any of the starches and polysaccharides sampled. Binding to different starches and polysaccharides was observed for SSI and SSIIa, and, to a lesser extent, for CBM53-3. Binding was below the detection limit for all linear and branched oligosaccharides tested, the only exception being 3-phosphomaltose, with the phosphate in the glucose at the reducing end. SSIIa, CBM53-3, and SSIV all showed strong binding to 3-phosphomaltose, while GBSSI showed weaker binding and binding was non-detectable for SSI.

Since maltose is an acceptor for glycosyl transfer we tested the ability of *Hv*SSIIIa to transfer glucose from ADP-Glc to 3-phosphomaltose by the spectrophotometric activity assay. When compared to maltose, transfer to 3-phosphomaltose was 17% higher.

### Coordinated production of SSs during barley endosperm development

**Figure 7:**
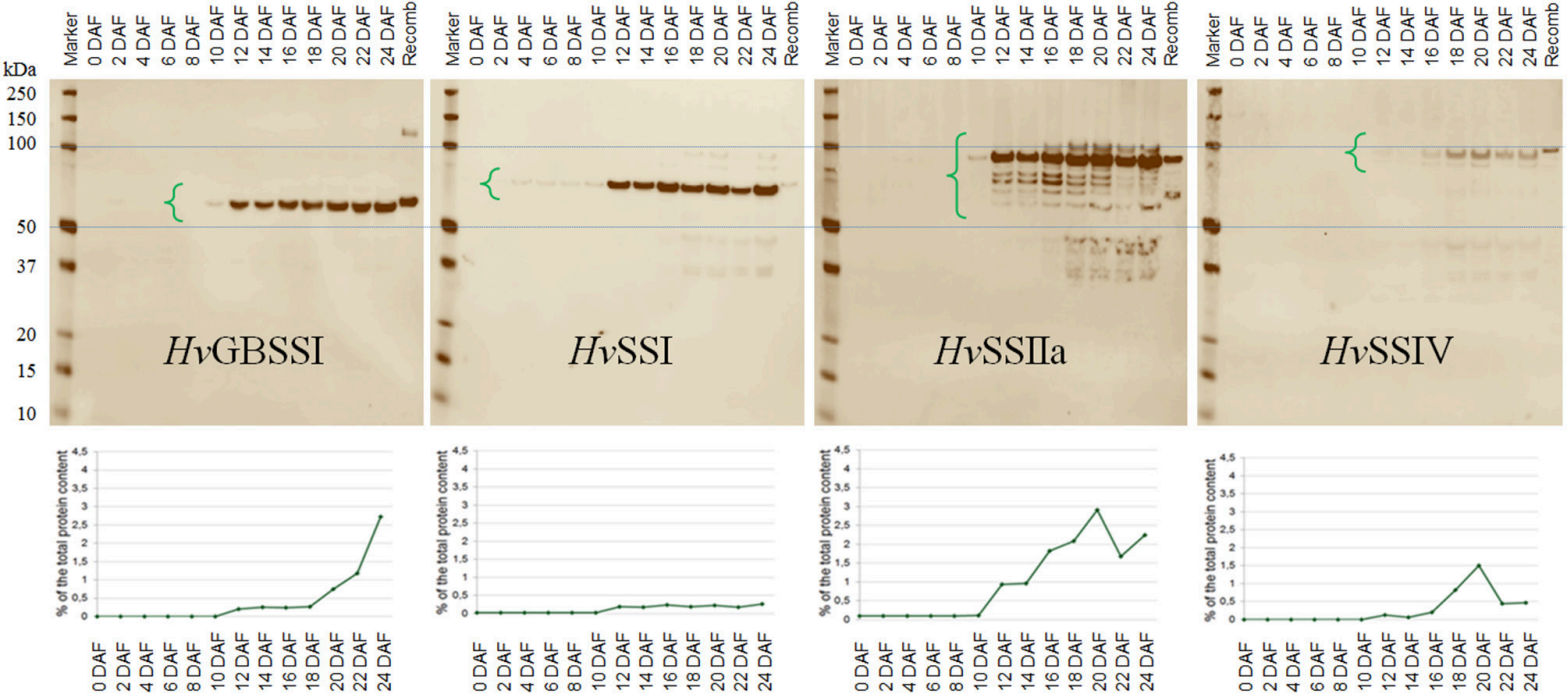
**Temporal profile of protein abundance in barley endosperm**. Barley grains were harvested between 0 and 24 DAF every 2 days and proteins from barley endosperm extracted. Equal amounts of protein were loaded onto SDS-PAGE and probed with the indicated primary antibodies. The horizontal blue lines show the alignment of the molecular weight markers around the relevant area. **(Top)** Immunoblots using the indicated antibodies; protein ladders are included in the left of each immunoblot and corresponding molecular weights are indicated to the left of the group. The rightmost lane in each blot shows recombinant enzyme controls added to each gel, of the same type that the antibodies were raised against. The molecular weights of the bands being integrated are: *Hv*GBSSI, 59.0 kDa (61.3 kDa for the recombinant version); *Hv*SSI, 67.5 kDa (69.8 kDa for the recombinant version); *Hv*SSIIa, 81.6 kDa (84.6 kDa for the recombinant version); *Hv*SSIV, 98.3 kDa (99.1 kDa for the recombinant version). The region used for integration in each case is illustrated with open green brackets; it is larger for SSIIa because the multiple bands were interpreted as being fragments of SSIIa, since their presence and pattern were occasionally detected also in SDS-PAGE of recombinant protein preparations; in any case, the band at 81.6 kDa dominates the overall intensity. **(Bottom)** Semi-quantitative analysis of protein abundance, ordered as for the immunoblots. The x-axis shows time as DAF, the y-axis shows protein quantity as calculated from immunoblot as percentage of total protein content.

The amount of barley SSs present between 0 and 24 DAF (days after flowering) was determined using semi-quantitative immunological analysis with highly purified recombinant SSs as standards (Figure 7). All SS and BE proteins were found in the insoluble fractions of the extract, which were used for quantification. No data were obtained for *Hv*SSIIIa and *Hv*SSIIIb as suitable antibodies were lacking. *Hv*BEI and *Hv*BEIIb were included for completeness (Figure S5). The level of cross-reactivity between the different SS proteins and anti-SS antibodies is illustrated in Figure S6. Our results show that protein levels of *Hv*GBSSI, *Hv*SSI, *Hv*SSIIa as well as *Hv*BeIIb drastically increase at 12 DAF. The protein levels of SSI remain constant until 24 DAF while those of *Hv*GBSSI continue to rise until 24 DAF. *Hv*SSIV protein production is not quantifiable before 12 DAF. Levels of *Hv*SSIV then stay relatively low until a peak appears around 20 DAF before they drop again. *Hv*BeI protein levels resemble those of SSIV, in that they start rising at 12 DAF, peak at 20 DAF, before they fall again. Overall, none of the tested proteins seems to be detectable before 4 DAF and only SSI is consistently detectable between 4 DAF and 12 DAF, although *Hv*GBSSI and *Hv*SSIIa are also visible at low level in that period. In average between 12 and 24 DAF, the least abundant protein of the ones tested is SSI (~0.21% of total protein) while the most abundant proteins are *Hv*SSIIa and *Hv*BeIIb (1.80 and 2.43% of total protein respectively). *Hv*GBSSI remains at relatively low levels until 18 DAF but becomes the most abundant protein at 24 DAF.

## Discussion

The analyses presented here aimed at measuring the properties of each SS involved in starch synthesis in barley endosperm. Consequently, only the isoforms GBSSIa and SSIIa, known to be responsible for storage starch synthesis, were produced and analyzed (Radchuk et al., 2009). Because it is unclear which one of the two SSIII isoforms is involved in endosperm starch synthesis in barley, we included both isoforms in this study. While Radchuk et al. identify both transcripts in barley endosperm (Radchuk et al., 2009), Hirose and Terao observed, in rice, the preferential transcription of one isoform in leaves and the other in endosperm (Hirose and Terao, 2004). Our activity profiles suggest that it is SSIIIb, in agreement with western blots using antibodies raised against peptides differing between SSIIIa and SSIIIb; we could detect a band for SSIIIb but not for SSIIIa in barley endosperm extracts. However the blots were of insufficient quality to be certain of the results.

### Acceptor substrate selection

We tested a SS from each class with all commercially available MOS, from glucose (DP = 1) to maltooctaose (DP = 8) (Figure 1). The natural acceptor substrates of the enzymes are, most of the time, the growing amylose and amylopectin molecules. However the results obtained with MOS are expected to reflect to some extent on substrate selection between the different non-reducing ends available in growing amylopectin. Furthermore, a certain amount of MOS is available at any time in the plastid environment, partly from starch degrading enzymes, and SSs can be expected to act on those as well. Lastly, MOS are likely to act as substrates at least during the initial stages of polysaccharide synthesis.

MOS substrate selection is similar for all SSs studied, with non-detectable activity for glucose and low but clearly detectable activity levels on maltose. This is in agreement with the results in *Arabidospsis* (Brust et al., 2013) indicating that the lack of activity on glucose and the presence of activity on maltose are likely to be general properties of all SS enzymes in all plants. Maltotriose (DP = 3) is a good acceptor for GBSSI, SSI, and SSII, but comparatively poor compared to longer MOS for SSIII and SSIV. The differences from DP = 4 to DP = 8 are minor with the exception of DP = 8 and GBSSI (discussed in further detail below). This is consistent with active site modeling in Cuesta-Seijo et al. (2013) and the structures of bacterial glycogen synthases (Sheng et al., 2009), where interactions with the acceptor are made with the three glucosyl moieties at the non-reducing end. The activity peak for *Hv*SSI at DP = 5 and the suggested peak at DP > 8 for *Hv*SSIIa are compatible with their effects in knockout plants (Morell et al., 2003; Zhang et al., 2004; Nakamura et al., 2005; Fujita et al., 2006), namely the preferential synthesis of chains with DP = 8–12 from chains of DP = 4–7 by SSI and of DP = 13–25 from DP ~ 10 by SSII. A slow increase in activity with acceptor length is only detected for SSIIa and SSIIIb, this is consistent with their supposed roles in elongation of B1 and B2 or larger chains of amylopectin (Jeon et al., 2010) and with data for bean SSIII (Senoura et al., 2007). The relatively flat activity profile for *Hv*SSIV is in contrast to the irregular pattern measured for *At*SSIV (Szydlowski et al., 2009). While we cannot exclude that this profile is species specific, that study (Szydlowski et al., 2009) shows a strong preference for DP = 3 both for SSIII and SSIV, which we do not observe for either enzyme. This might be an experimental artifact related to the use of phosphorylase for modification of products in Szydlowski et al. (2009) since phophorylase is reversible and does not favor DP = 3 as a substrate. On the contrary, the profile measured here for *Hv*SSI is very similar to that measured for *At*SSI (Delvallé et al., 2005; Skryhan et al., 2015).

A direct comparison between the activities with MOS and branched polysaccharides is difficult as the number and accessibility of non-reducing ends is different and often unknown, but the relative variations can be enlightening. We find that barley SSs can be divided into two groups: *Hv*GBSSI, *Hv*SSI, *Hv*SSIIa and *Hv*SSIIIb display higher activity with polysaccharides (tested at 1 mg/mL) than with MOS (tested at 10 mM); in contrast *Hv*SSIIIa and *Hv*SSIV, have lower activities with polysaccharides compared to MOS acceptors. The specific activities measured for GBSSI and SSIIIa were relatively small. It is likely that the activity of *Hv*GBSSI would be enhanced inside granules and we cannot discard that the profile of activity might differ in that environment (Denyer et al., 1999a). Similarly, we cannot discard that SSIIIa is lacking important modifications or binding partners in our *in vitro* assays that could affect its level of activity.

SSIV displays a particularly high level of activity on MOS together with a particularly low, close to the detection limit, level of activity on polysaccharides. This suggests a scenario where SSIV is not involved in the elongation of amylopectin but rather in the synthesis of long MOS chains, something which was implied in Szydlowski et al. (2009) but which is made explicit by comparison with all other SSs. Such long MOS chains could serve as seeds for amylopectin synthesis initiation which would explain the key role played by SSIV in granule initiation (Szydlowski et al., 2009; Ragel et al., 2013). Long MOS chains could as well simply diffuse to preexisting granules where they could contribute to the growth of amylopectin via the action of branching enzymes.

None or low activity was measured with a panel of alternative acceptor substrates including glucose and small branched or modified linear oligosaccharides, making it unlikely that they have a significant role as substrates *in vivo* (Figure S1). The exception was maltosyltrehalose, which features a linear maltotriosyl moiety at its non-reducing end. This confirms the maltotriosyl unit as the minimal unit effectively recognized as substrate by SS enzymes. Since maltosyltrehalose does not have a reducing end, this adds to the existing body of evidence that SS activity does not involve the reducing end of glucosyl substrates. Branched substrates including a maltosyl unit at the non-reducing end showed activities similar to those with maltose, with the 1,6-glycosydic bond having little or no effect.

SS enzymes are normally considered to be involved purely in the elongation of glucan chains, and to be limited in the extension of that elongation with the possible exception of GBSS. Through our *in vitro* analysis we found both assumptions to be misleading. Brust et al. (2013) detected a reverse reaction with *At*SSI. A similar experiment performed here with *Hv*SSI yielded similar results, with *Hv*SSI being able to use ADP to shorten glucan chains with concomitant production of ADP-Glc. The reverse reaction is much slower and quantifying exactly by how much is problematic. If ADP is non-saturating at physiological concentrations it would be more than 100 times slower than the forward reaction, in rough agreement with a value of Δg^0^ of −11.7 kJ/mol for the elongation reaction estimated with the eQuilibrator web server (Noor et al., 2013). This reversibility is thus of limited biological significance, but it might be significant in *in vitro* experiments where donor ADP-Glc is depleted, in which case re-mobilization of glucose monomers by the action of SSs can be expected. We don't know whether other SSs also catalyze the reverse reaction, although traces of it are detected in other experiments reported in this paper, but this is to be expected to be the case from thermodynamic considerations.

Extension experiments with SSI, surprisingly, allowed us to quantitate products up to DP = 43 and detect significantly longer MOS. Thus, there seems to be no limit to the length of products created by the action of *Hv*SSI *in vitro*. This is in direct contradiction with the results of Commuri and Keeling (2001), where it was argued that the affinity of maize SSI for acceptors increased exponentially as DP approached 20 to the point of SSI becoming entrapped and inactivated. It must be noted that this conclusion was based on two data points which in our opinion are not consistent with the methods used to derive them, both of which involve divisions by very small numbers and thus prone to large errors. Also, the exponential affinity assumption is contradictory with the activities reported there on the very same substrates.

In some measurements we used a truncated version of *Hv*SSI and a modified acceptor substrate, but we propose the results to be of general validity. The fact that the reverse reaction mostly stops at DP = 3 indicates that the fluorescently labeled acceptor is recognized as a DP = 6 molecule by *Hv*SSI. The truncated version of SSI employed was chosen merely because of its speed and its properties resemble those of the rice enzyme (Cuesta-Seijo et al., 2013) and of naturally truncated maize SSI (Imparl-Radosevich et al., 1998). Additionally, a time series carried out with wild type *Hv*SSI (Figure 2C) is also consistent with the walking Gaussian distribution of products observed in the donor-limited experiments.

Our results suggest that *Hv*SSI is incapable of discriminating between acceptor chains of DP > 7, using them all equally. This seems at first contradictory with the fact that the lack of SSI activity in plant mutants results in a reduction of chains around DP = 8–10 in particular (Delvallé et al., 2005; Fujita et al., 2006). Why does it not affect longer chains as well? Our experiments are done in isolation. *In planta* there is competition between the different SSs, both for acceptors and donors. The effect observed *in planta*, largely limited to the DP 6–12 range, is likely the consequence of SSI slightly favoring chains with DP around 5 and SSII slightly favoring longer chains, possibly around DP = 12, which means that their action concentrates preferentially, but not exclusively, in such acceptor chains. If we speculatively argue that the relative rates only allow SSI to act, in average, five times per chain, the mutant results can be explained without the need for an elongation limit for SSI.

Elongation of MOS by SSI until donor is exhausted leads in our hands to very reproducible distributions of products controlled simply by the acceptor/donor ratio. We thus propose this as a method for the *in vitro* production of well-defined MOS distributions for studies of other enzymes. This might offer advantages compared to the use of phosphorylase (Nakai et al., 2013; O'Neill et al., 2014), which while being faster, catalyzes a highly reversible reaction.

### Distributive vs. processive or dual mechanisms of action

GBSSs from potato and pea were reported to behave like distributive enzymes in the absence of an amylopectin matrix but as processive enzymes in the presence of amylopectin (Edwards et al., 1999; Denyer et al., 1999a). In those experiments, when unlabeled DP = 3 was extended with a small amount of radioactively labeled ADP-Glc, the only detectable product was DP = 4 in the absence of amylopectin, while longer products were formed from DP = 3 in the presence of amylopectin. We analyzed the behavior of *Hv*GBSSI strictly in the absence of any polysaccharide matrix. For very small reaction advances we also observe the formation of DP = 4 only. This is consistent with a distributive mechanism, an inference which is extended by fit to simulation up to DP = 6 (Figure 3B). The behavior of GBSSI then changes, even in the absence of any starch matrix. Products of DP = 7 start to accumulate even when DP = 6 and DP = 8 do not. At the same time, small amounts of species of DP = 8–20 accumulate rapidly in a manner much less sequential than what would be expected from a distributive model based on simulations. The same behavior is observed at a qualitative level in reactions starting from DP = 8. This behavior is consistent with a model in which, starting with DP = 8, products are not always released from the enzyme. Instead they are often retained and extended for a further reaction cycle, that is, a processive mode of action *in vitro*. The probability of such processive events seems to be no larger than 50%, so that very long reaction sequences would be rare. Further data would be needed for a more detailed interpretation. This dual mechanism of action would also explain the large increase in activity observed for DP = 8 in our spectrophotometric assay. Each reaction, possibly consisting of several consecutive cycles, would effectively result in more than one molecule of ADP-Glc consumed and thus a higher apparent reaction rate.

The mechanism of action of all soluble SS enzymes is commonly accepted to be purely distributive, with complete release of products after each reaction cycle. Our experimental *in vitro* data for SSI, SSIIa, SSIIIb, and SSIV correspond to this model, with an almost perfect fit between experiment and simulation for SSI, SSIIa, and SSIIIb when a simple kinetic model is fed with the experimental reaction velocities measured for each acceptor species (Figures S3E,H,I). The fit for SSIV is less perfect than for the other enzymes. In the absence of further data, we abstain from making any mechanistic claims, but presence of products with a high degree of polymerization would be qualitatively compatible with a certain degree of processivity as explained for GBSSI, which we can thus not discard.

### Effects of temperature

Protein stability was assessed by means of incubations at different temperatures. This produced *in vitro* unfolding midpoints between 39.8 and 47.4°C, with all proteins tested showing close to 100% stability at least up to 36°C. The lower thermostability of GBSSI can probably be explained by the absence of its natural environment inside the granule, while the SSIIa sample used in this experiment had only ~50% purity as estimated by SDS-PAGE, so that unfolding of protein impurities might have had a negative effect. Extra stabilization is measured in the presence of extra sugars in the solution, which would provide further stabilization in the plastids from the starch already present. Thus, all barley SSs had thermostability in excess of the expected needs in growing fields, and should not be inactivated by elevated temperatures in their natural environment.

The activity of *Hv*SSI, *Hv*SSIIa, and *Hv*SSIV increased with temperature for all MOS substrates tested from maltose to maltooctaose, reflecting the expected behavior of any chemical system before structural stability becomes limiting. In the case of *Hv*SSI the results obtained with glycogen and soluble starch are in contrast to those obtained for MOS substrates. The activity with glycogen peaks at 30°C with a strong and steady decline of several-fold from there up to 40°C. Since the activity with MOS is not compromised at the highest temperatures, we can discard this being the effect of structural de-stabilization of the enzyme. One possible explanation is that certain conformational features in the substrate itself, possibly an arrangement on terminal chains relative to branching points or the partial formation of double helices of glycan, is affected by temperature yielding a less optimal substrate for SSI at higher temperatures. An alternative explanation involves the disruption of the high affinity surface binding site for maltopentaose and larger sugars (Cuesta-Seijo et al., 2013; Wilkens et al., 2014). If this binding site, which includes a surface loop, is affected by elevated temperatures, it would result in reduced local concentration of SSI on the surface of glycogen and amylopectin molecules and thus a reduced reaction rate at higher temperatures. We did not test at temperatures below 27°C, but the presence of a plateau of SS activity on polysaccharides at 27–30°C together with the behavior with MOS suggest that the activity on polysaccharides is likely to drop at temperatures below 27°C. This would be in agreement with results in rice and potato starch synthesis where a phosphorylase based pathway became increasingly important at temperatures below 20°C (Satoh et al., 2008; Fettke et al., 2012).

### Binding affinities from microarray data

Two trends are apparent in our data. One, SSI and SSII are bound to a large series of starches and polysaccharide types with moderate affinity, which is in agreement with existing data (Mu-Forster et al., 1996; Fujita et al., 2006; Stensballe et al., 2008) although this might also be mediated by association into multiprotein complexes (Hennen-Bierwagen et al., 2008; Tetlow et al., 2008). Similarly, SSIII is expected to be bound to starch (Senoura et al., 2007; Valdez et al., 2008). We can detect weak binding from the CBM53-3 domain alone, while CBM53-2 is expected to show a higher affinity for starch than CBM53-3 (Valdez et al., 2008). A possible explanation for the lack of GBSSI binding to starch is that it is bound so strongly or buried deep enough as to reduce the accessibility of antibodies to the His-tag. Alternatively, the lack of a recently identified protein involved in transporting GBSS to the interior of granules (Seung et al., 2015) might be inhibiting its interaction with starch. The lack of detected binding to starch and polysaccharides by SSIV is compatible with the role in extension of MOS that we are suggesting and with its low level of activity on polysaccharides. The lack of binding of all SSs to linear and branched neutral oligosaccharides simply indicates that the binding is weak or reversible enough as to be below the detection limit.

Our dataset identifies a strong affinity of SSIIa, SSIII, and SSIV for 3-phosphomaltose. This substrate mimics the 3-phosphorylation of starch found *in vivo*, an important helix breaker in crystalline starch (Blennow and Engelsen, 2010). Since the activity of SSIIIa, which includes the CBM53-3 domain, is not significantly higher on 3-phosphomaltose than on maltose, the effect of this phosphorylation could be to navigate SSII, SSIII, and SSIV (but not SSI) to phosphorylated positions. This suggests a possible stimulating effect of 3-phosphorylation already during starch biosynthesis as recently suggested (Skeffington et al., 2014).

### Kinetic parameters and comparison of activity levels

Obtaining K_M_ constants for varying amounts of ADP-Glc was relatively straightforward. K_cat_ values were more problematic and only provided lower boundary values as already described in the results section. The highest and lowest donor affinities corresponded to K_M_s in the order of 0.1 and 0.6 mM for GBSSI and SSI respectively, with intermediate values for SSIIa, SSIIIb, and SSIV. Most of the measured K_M_ values for ADP-Glc are similar to those measured for enzymes from other plants, but the value measured for SSI is more than twice as high as other reported values (Macdonald and Preiss, 1985; Imparl-Radosevich et al., 1998, 1999a; Cao et al., 2000; Gao et al., 2004; Senoura et al., 2004, 2007) with the exception of an even higher value recently reported by Nakamura et al. (2014). This probably correlates with the fact that in barley, SSI is not the dominant SS activity (Morell et al., 2003), in contrast to other cereals or plants (Peng et al., 2001; Delvallé et al., 2005; Fujita et al., 2006). While the measured K_M_ of *Hv*GBSSI for ADP-Glc is comparable in magnitude to values reported for GBSS in maize (Macdonald and Preiss, 1985), they are one order of magnitude smaller than values previously reported for GBSS from potato (Edwards et al., 1999) and pea (Clarke et al., 1999). The reasons for this are unknown but it might simply reflect inter-species variations. The values for SSIII are intermediate between those measured for the full protein and the catalytic domain of SSIII in *Arabidopsis* (Busi et al., 2008; Valdez et al., 2008; Wayllace et al., 2010).

Only in some cases was it possible to fit a Michaelis-Menten model for varying concentrations of MOS acceptors; saturation was not achieved with SSI, SSIIa and SSIIIb. The lowest K_M_ value was for SSIV: 20.4 mM with DP = 5. This value is half of that measured for GBSSI, while a study on GBSSI from pea failed to measure any saturation with DP = 3 up to 1 M concentrations (Denyer et al., 1999a). Parallels with GBSSI, for which linear MOS are the natural substrate, and that the affinity of SSIV for MOS is at least an order of magnitude larger than for SSI, SSIIa, or SSIIIb, further supports the notion that MOS are the natural substrate of SSIV.

In this study we also analyzed the abundance of the different SSs in barley endosperm (together with pericarp), quantifying it in two day intervals from 0 to 24 DAF. Our results roughly, but not exactly, correlate with the levels of gene expression reported by Radchuk et al. (2009). Combining this data with the specific activities already discussed, *Hv*SSIIa would be the most active SS enzyme from its point of detection at 12 DAF until 24 DAF, in agreement with Morell et al. (2003). This is of course with the possible exception of SSIIIb, present in unknown amounts. The amount of *Hv*SSI measured is several-fold lower from 12 DAF on, so that even with a higher specific activity on some substrates its activity will be lower than that of SSIIa. This is in contrast with the results obtained in other cereals, with SSI playing a dominant role in rice (Fujita et al., 2006), maize (Cao et al., 1999), and wheat (Li et al., 2000). SSI is the only SS enzyme that was present in amounts enough to be quantified before 12 DAF. This might correspond to SSI enzyme present in the pericarp, consistent with the transcript levels observed by Radchuk et al. (2009). This suggests that transient starch synthesis in the pericarp might be dominated by SSI, and possibly also by SSIIb which also has high transcript levels in the pericarp and which was not quantified here. SSIV, which we are proposing is more active on MOS substrates than on amylopectin, might temporarily be the most active protein (MOS substrates only) around 20 DAF. It is intriguing that SSIV, supposedly involved in granule initiation, is not detectable before 12 DAF. While expression at or close to 12 DAF is compatible with a role in starch initiation in endosperm, it is not compatible with initiation of starch production in the pericarp. This would argue in favor of an involvement of other proteins in that function, but it must be noted that, with its high specific activity on MOS, very small amounts of SSIV could suffice to create a pool of MOS large enough to initiate the formation of branched polysaccharides by branching enzymes. The large level of activity of SSIV on maltose would make maltose a suitable candidate as an initiator molecule if significant amounts could be transported into the amyloplast, for example through a maltose transporter (Niittylä et al., 2004), although such transporter has never been identified in endosperm.

It must be emphasized one last time that this data originates from purified enzymes studied *in vitro*. While our data should be free of the effects of contaminants or pleiotropic effects, the activity of these enzymes *in vitro* might differ in certain aspects from the activity *in vivo*, where the effect of different environments like the granule matrix itself, post-translational modifications like phosphorylation (Glaring et al., 2012; Momma and Fujimoto, 2012), redox processes (Skryhan et al., 2015) or complex formation (Hennen-Bierwagen et al., 2008; Tetlow et al., 2008; Liu et al., 2012) will affect protein activity. Thus, all conclusions or insights derived from it should be validated *in planta*. The data presented here will hopefully serve to guide efforts to understand the different roles played by SSs in cereal endosperm synthesis.

## Author contributions

JC, MN, CR, and MP conceived and designed the experiments. SB, MM, and WW designed and provided critical materials and assays. JC, MN, CR, KK, SB, MR, YY, and AS prepared the materials and performed the experiments. All authors contributed to the writing of the paper.

## Funding

This work was supported by the Carlsberg Foundation and by Danish Strategic Research Council and The Danish Council for Independent Research, Technology and Production Sciences as part of the GlycAct project (FI 10-093465).

### Conflict of interest statement

The authors declare that the research was conducted in the absence of any commercial or financial relationships that could be construed as a potential conflict of interest.
