# Supplementary material for: In vitro Biochemical Characterization of All Barley Endosperm Starch Synthases
Source: Front Plant Sci. 2016 Jan 28;6:1265. doi: 10.3389/fpls.2015.01265 (PMC4730117; doi:10.3389/fpls.2015.01265)
Supplement: Supplementary file 1 [file SupplementaryInformation.PDF]

Supplementary Material

In Vitro Biochemical Characterization of All Barley Endosperm Starch Synthases

Author list: Jose A. Cuesta-Seijo[1*], Morten M. Nielsen[1], Christian Ruzanski[1,2], Katarzyna Krucewicz[1], Sophie R. Beeren[1,3], Maja Gro Rydhal[4], Yayoi Yoshimura[1,5], Alexander Striebeck[1], M. S. Motawia[4], William G. T. Willats[4] and Monica M. Palcic[1,6]

Affiliations:
[1]Carlsberg Laboratory, Copenhagen, Denmark.
[2]Novo Nordisk A/S, Måløv, Denmark.
[3]Department of Chemistry, Technical University of Denmark, Kgs. Lyngby, Denmark.
[4]Department of Plant and Environmental Sciences, University of Copenhagen, Frederiksberg, Copenhagen, Denmark.
[5]Division of Molecular Science, Faculty of Science and Technology, Gunma University Tenjin-cho, Kiryu, Gunma, Japan.
[6]Department of Biochemistry and Microbiology, University of Victoria, BC, Canada.

[*] **Correspondence:**
Jose Antonio Cuesta-Seijo
Carlsberg Laboratory
Gamle Carlsberg Vej 10
DK-1799 Copenhagen V,
Copenhagen, Denmark
josea.cuesta.seijo@carlsberglab.dk

Estimation of reaction rates based on product size distribution:

To estimate the reaction rates from the product distribution resulting from the reaction of the fluorescently labelled DP = 6 substrate with 15 equivalents of ADP-Glc, a simple kinetic simulator was built in Microsoft Excel. The rates of the elongation reactions were considered to increase linearly with the amount of each acceptor substrate present at any given time point. This assumption holds well at concentrations of acceptor well below K_M, which is a proven assumption for MOS of DP < 8 and a reasonable assumption for longer MOS in the distribution considering that the total MOS concentration was 0.1 mM.

Starting with a molar fraction of 1.0 for DP = 6, the production of longer MOS was followed in small reaction steps which were defined in arbitrary reaction progress units rather than in time units. Steps of 10% of each acceptor used per step, multiplied by the relative reaction velocities for each acceptor, were used. The results differed little from those using smaller reaction steps. Avoiding time units in this way, only the relative velocities of each forwards reaction step need to be considered. In each step of the simulation, the amount of each acceptor used and the amount of each product with DP increased by 1 were calculated, and the amounts of each MOS present updated accordingly.

An arbitrary velocity value of 1.0 was assumed for acceptors of DP = 6 and DP = 7 with a relative reaction velocity of 0.73 for DP = 8, these velocities being derived from our experimental measurements with those substrates. For acceptors longer than DP = 8 for which no experimental data was available, models in which the reaction velocity was reduced by a constant factor with each elongation step were created. For example, a 3% decay model would have, for DP = 9, 97% of the reaction velocity for DP = 8; while DP = 10 would have a velocity 97% of that for DP = 9. In that case the relative reaction velocities used would be 0.73, 0.708 and 0.687 for DP = 8, 9 and 10, respectively, resulting for example in a relative velocity of 0.435 for DP = 25 and so on. The no decay model was simply a model with 0% decay, resulting in relative velocities of 0.73 for all acceptors with DP ≥ 8. The calculations were limited to DP = 35.

This simulation ignores the presence of the reverse reaction, which was considered to be sufficiently slow to have a negligible effect. In the presence of a much faster forwards reaction, its effect is likely to be just a broadening of the product distribution resulting from reverse reaction cycles being neutralized by the faster forwards reaction cycles.

The final product distribution was taken at the calculated reaction progress point where the average DP matched that of the experimentally measured distribution.

Distributive mechanism of action of soluble starch synthases.

The product profile for SSI starting from DP = 3 with 1.5 eq. of ADP-Glc was measured for SSI (Fig. S3a), it shows excellent agreement with that predicted based on a purely distributive mechanism (Fig. S3e). For SSIIa the reactions with DP 3 and DP 8 as starting acceptors and 1.5 equivalents of ADP-Glc were followed over time. Protein activity was too low to allow the reaction to proceed to completion, but it proceeded to a significant amount nevertheless. Starting from DP = 3 it can be seen that initially only DP = 4 is formed and only after DP = 4 starts to accumulate does DP = 5 start to form (Fig. S3b). Products with DP = 6 and DP = 7 are only formed in the latest time point and in small amounts. The predicted product distribution, calculated as for SSI with a purely distributive model, is in excellent agreement with the experimentally measured distribution (Fig. S3f). A similar result was obtained with SSIIa starting from acceptors of DP = 8 (Fig. S3c), where the commercial sample used was found to be contaminated with significant amounts of DP = 7 and smaller MOS. Products of DP = 9, 10, 11, 12 and 13 are formed strictly in sequential order. We do not have data on individual reaction velocities for acceptors of DP > 8, but the experimental distribution matches the calculated one if we assume an increase in reaction velocity by a factor of 1.5 for each extra glucosyl unit (Fig. S3g). Only end points after 24 hours were measured for SSIIIb and SSIV (Fig. S3a), but in both cases the data match the predicted product distributions at least at a qualitative level (Figs. S3h,i) if we assume constant reaction velocities for DP > 8; although the reaction products of SSIV deviate to a larger degree than those for

other enzymes, including peaks extending to DP = 14 with diminish in area slower than we can predict with any distributive reaction model. This group of peaks at large DP which diminish in area in an approximately linear fashion resembles that observed for GBSSI starting from DP = 3 (Fig. 3) and from DP = 8 (Fig. S3d,j).

Amino acid sequences for all used proteins:

Barley granule-bound starch synthase (*Hv*GBSSI, from Genbank accession: AAM74048):

MGSSHHHHHHSSGLVPRGSHMSATGSGMNLVFVGAEMAPWSKTGGLGDVLGGLPPAMAANG
HRVMVVSPRYDQYKDAWDTSVISEIKVADEYERVRFFHCYKRGVDRVFIDHPWFLEKVRGKTK
EKIYGPDAGTDYEDNQQRFSLLCQAALEAPRILNLNNNPYFSGPYGEDVVFVCNDWHTGLLAC
YLKSNYQSNGIYRTAKVAFCIHNISYQGRFSFDDFAQLNLPDRFKSSFDFIDGYDKPVEGRKINW
MKAGILQADKVLTVSPYYAEELISGEARGCELDNIMRLTGITGIVNGMDVSEWDPTKDKFLAVN
YDITTALEAKALNKEALQAEVGLPVDRKVPLVAFIGRLEEQKGPDVMIAAIPEILKEEDVQIILLG
TGKKKFEKLLKSMEEKFPGKVRAVVRFNAPLAHQMMAGADLLAVTSRFEPCGLIQLQGMRYG
TPCVCASTGGLVDTIVEGKTGFHMGRLSVDCNVVEPADVKKVATTLKRAVKVVGTPAYQEMV
KNCMIQDLSWKGPAKNWEDVLLELGVEGSEPGIVGEEIAPLAMENVAAP

Barley starch synthase I (*Hv*SSI, from Genbank accession: AAF37876.1):

MGSSHHHHHHSSGLVPRGSHMARLRRVARGRYVAELSREGPAARPAQQLAPPVVPGFLAPPPP
APAQSPAPTQPPLPDAGVGELAPDLLLEGIAEDSIDTIVVAASEQDSEIMDANDQPLAKVTRSIVF
VTGEAAPYAKSGGLGDVCGSLPIALAARGHRVMVVMPRYLNGTSDKNYAKALYTGKHIKIPCF
GGSHEVTFFHEYRDNVDWVFVDHPSYHRPGSLYGDNFGAFGDNQFRYTLLCYAACEAPLILEL
GGYIYGQSCMFVVNDWHASLVPVLLAAKYRPYGVYRDSRSTLVIHNLAHQGVEPASTYPDLGL
PPEWYGALEWVFPEWARRHALDKGEAVNFLKGAVVTADRIVTVSQGYSWEVTTAEGGQGLNE
LLSSRKSVLNGIVNGIDINDWNPTTDKCLPHHYSVDDLSGKAKCKAELQRELGLPVREDVPLIGF
IGRLDYQKGIDLIKMAIPDLMREDVQFVMLGSGDPVFEGWMRSTESSYKDKFRGWVGFSVPVS
HRITAGCDILLMPSRFEPCGLNQLYAMQYGTVPVVHGTGGLRDTVETFNPFGAKGEEGTGWAFS
PLTVEKMLWALRTAISTFREHKPSWEGLMKRGMTKDHTWDHAAEQYEQIFEWAFVDQPYVM

Barley starch syntase IIa (*Hv*SSIIa, from Genbank accession: AAN28309):

HMGHHHHHHHHHHSSGHIDDDDKHMAARAAGIDDAAPGRQPRARRYGAATKVADPVKTLDR
DAAEGGGPSPPAPRQDAARLPSKNGTLINGENKPTGGGGATKDSGLPTPARAPHLSIQNRVPVN
GENKHKVASPPTSIVDVASPGSAANISISNKVPPSVVPAKKTPPSSVFPAKKTLPSSGSNFVSSASA
PRLDTVSDVELAQKKDALIVKEAPKPKALSAPAAPAVQEDLWDFKKYIGFEEPVEAKDDGSAV
ADDAGSFEHHQNHDSGPLAGENVMNVVVVAAECSPWCKTGGLGDVAGALPKALAKRGHRVM
VVVPRYGDYEEAYDVGVRKYYKAAGQDMEVNYFHAYIDGVDFVFIDAPLFRHRQQDIYGGSR
QEIMKRMILFCKAAVEVPWHVPCGGVPYGDGNLVFIANDWHTALLPVYLKAYYRDHGLMQYS
RSVMVIHNIAHQGRGPVDEFPFTELPEHYLEHFRLYDPVGGEHANYFAAGLKMADQVVVVSPG
YLWELKTVEGGWGLHDIIRQNDWKTRGIVNGIDNMEWNPEVDVHLKSDGYTNFSLKTLDSGK
RQCKEALQRELGLQVRGDVPLLGFIGRLDGQKGVEIIADAMPWIVSQDVQLVMLGTGRHDLES
MLQHFEREHHDKVRGWVGFSVRLAHRITAGADALLMPSRFEPCGLNQLYAMAYGTIPVVHAV
GGLRDTVPPFDPFNHSGLGWTFDRAEAHKLIEALGHCLRTYRDHKESWRGLQERGMSQDFSWE
HAAKLYEDVLVQAKYQW

Barley starch synthase IIIa (*Hv*SSIIIa, from Genbank accession: AEL97583):

EGDIHMVKVISSRGYTTRLIVEPSTENTEHNNGDEEILDTYNALLHTEAAEWTDTREAETAEADS
SQNASSSSIIRELDAADEDILAVDLTVNALSSVTKREVDAADKARVEEDVFELDLPATTLRSVIVD
VIDDNGTVQETLRSVIVDVTDDVADKARVEEDIFELDFSGNVSSSATTVELDAVDEVGPVQDTFE

ASLSGNVSNSATVQEVDAVDEAGNDQDIFKADLSGNIFSSSTTVEVGVVDEAGSTKDRFEMDSS
GNVSTSATMWDAIDEAVADQDAVEADLSGNASSWATYRELDDMVDENRSEEETFVMDLVGEA
TDEEENYQQQYPVPSSFSMWDKAIAKTGVSLNPEPRLTSVQEQGKVNFSDKQDLSIADLPGQNQ
SIVGSCKQDKSIADVAGPPQSIFGSSKQHRPIVAFRKQNHSIVSDPKQKQSIVGFRSQDLSAVDLP
KQNIPIVGTSSEGQTKQVPVVDRQDALYVNGLELKEGDHRSEKTDEDALHVKFNIDNVLQKHLA
DRTQAVETTIWKEVDEEHLYMTEHQIGSTEGHMVLNEDELSITEIGMGSGDKIQHALSEEELSWS
EDEVQLNKDDGQYEVDETSASFTVEQDIQGPPQDVVDPQALRAMLQELADKNYSMRNKLFVFP
EVVKADSVIDLYLNRDLTALANEPDVVIKGAFNGWKWKLFSERLHKSDLGGVWWSCKLHIPKE
AYRLDFVFFNGRTVYENNGNNDFCIGIEGTMNEDLFEDFLVKEKQRELEKLAMEEAERRTQTDE
QRRRKEARAADEAVRAQAKAEIEIKKNKLHSMLSLARTCDDNLWYIEASTDTRGDTIRLYYNR
NSRPLAHSTEIWMHGGYNNWTDGLSIVESFVKCNDEDGDWWYADVILPEKALVLDWVFADGP
AGNARNYDNNSRKDFHAIIPNKNVTKKGFWVQEEQNIYTRLLQERREKEEIMRRKAERSANMK
AEMKAKTMRRFLLSQKHIVYTEPLEVRAGTAVDVLYNPSNTVLNGKTEVWFRCSFNLWMHPS
GALPPQKMVKSGDGSLLKATVNVPPDAYMMDFVFSESDENGIYDNRNGMDYHIPVSDSIETEN
YMRIIHIAVEMAPVAKVGGLGDVVTSLSRAVQDLGHTVEVILPKYDCLNQSSVKDLHLYQSFSW
SGTEIKVWVGQVENLTVYFLEPQNGMFGVGCVYGRNDDRRFGFFCHSALEFILQNGLSPHIIHCH
DWSSAPVAWLYKEHYSQSRMASTRVVFTIHNLEFGAHYIGKAMTYCDKATTVSPTYSREVAGH
GAIAPHREKFYGILNGIDPDIWDPYTDNFIPVPYTYENVVEGKSAAKRALQQKFGLQQTDVPVV
GIITRLTAQKGIHLIKHAIHQTLESNGQVVLLGSAPDHRIQGDFCRLADALHGVYHGRVKLVLTY
DEPLSHLIYAGSDFILVPSIFEPCGLTQLVAMRYGSIPIVRKTGGLHDTVFDVDNDKERARSLGLE
PNGFSFDGADSNGVDYALNRAIAAWFDARDWFHSLCKRVMEQDWSWNRPALDYIELYHAARK
FHHHHHH

Barley starch synthase IIIb (*Hv*SSIIIb, from Genbank accession: AFI61839):

MGRPHHHHHHGKPIPNPLLGLDSTENLYFQGSAPRPSAESWTSTKKIEHGSADEDGDLGRSNGT
LSGDATEQASTAEEESEVEFAGNVSSSAGLEGVAETEKEADQNQLPALSSTSMDVESVNEATEQ
TIIGDESSVVDFSGNVSGSVALEVVDESETEEEADQKQSSSLASTSMDSESIDRELEGYRARINAL
VGASTQEQDRSIIGVHDQDKSVISPNEQDISAVDVPEQGLSGVGVTREDPTKQTTSKQEITEAAVE
EISSKNFVARKHSISGDGVEASKNANQQPLVDGDELKITEDEEQHEPEMQEYVRKDVDLQALKR
RLEELADGNYSIGNKCFVFPELVKADSVIDLYLNRSMSALASEPDILIKGAFNGWRWNHFTERLH
KSELSGDWWCCKLYIPKQAYRLDFVFFNGDTVYENNSYNDFVLHIESDIDEHSFEDFLLEEKQRE
LERLAAEEAERERQAEEERRKEEERAAMEADRAQAKAEVEMTRNNLQHVLGLASRYANNLWY
IEPNTYKGGDRVRLYYNRSSGPLKHKNEIWLHGSYNNWTDGPSIVERLVKSDEQDGDWWYAN
VTLPESALVLDWVFADGPPGNARKYDNNGKRDFHAAVSKSISEDLFWVKEEHRIFERLQRERKE
KEDAGRRKAEITARMKAEMKEKTMREFLLSQKHIVYTEPLEVRAGMTVDVLYNPSNTVLNGSP
EVWFRCSFNRWTHPSGPLPPQKMVNAENGSHLQATVRVPLDAYMMDFVFSESEEGGMYDNRD
GMDYHIPVSDSMAKEPPMHIVHIAVEMAPIAKVGGLGDVVTSLSRAIQDLGHKVEVVFPKYDCL
NLSNVKDLHCRQSFTWGGTEIKVWFGKVEGISVYFLEPQNGMFWVGCVYGRNDESRFGFFCHS
ALEFLLQSGSSPDIIHCHDWSSAPVAWLYKQHYVHNGLPNGRVVFTIHNLEFGVHHIGKAMAHC
DKATTVSYTYSKEVSGHGSIAPHYFKFHGIRNGIDSDIWDPYNDNFIPVHYTSENVVEGKSSAKR
ALQEKLGLHQTDSPLVGIISRLTAQKGIHLIKHAIYRTLERNGQVVLLGSAPDHRIQGDFSNLASK
LHGEFDGRVKLCLTYDEPLSHLIYAGADFILVPSIFEPCGLTQLTAMRYGSIPIVRKTGGLYDTVF
DVDDDKDRAREQGLEPNGFSFEGADSNGVDYALDRAITTWYDARGWFHSLCKRVMEQDWSW
NRPALDYMELYHSARKN

Barley starch synthase IV (*Hv*SSIV, from Genbank accession: BAJ86666):

AAAQQRPQRSTAGADHLNTAANVRSDEAAVSAEKERQRKYSDGDGISNLQLEDLVEMIQNTEK
NILLLNQARLQAMERADKILKEKEALQRKISILETRLSETDAQHKLSSEGNFSESPLALEFDVLKE
ENILLKEDIEFFKSKLIEVAKTEEGIFKLEKERALLDASLRELESRFIAAQADMMKLGPRDAWWE
KVEKLEDLLETTANQVERAAVILDNNHDLQDKLDKLVASLQAANISKFSSSLVDLLQQKVKLVE
ERFQACNREMHSQIKLYEHSIVEFHDTLSKLIEESEKRSLENFTGNMPSELWSKISLLIDGWLLEK

KISYNDASLLREMVRKRDSRLREAYLSYRGTENREVMDNFLKMALPGTSSGLHIAHIAAEMAPV
AKVGGLADVISGLGKALQKKGHLVEIILPKYDCMQVDQVSNLKVLDVPVQSYFEGNMFDNKIW
TGTVEGLPVYFIEPQHPAMFFSRAQYYGEHDDFKRFTYFSRAALELLYQSGKKVDIIHCHDWQT
AFVAPLYWDVYANLGFNSARICFTCHNFEYQGTAPARDLAWCGLDVEHLDRPDRMRDNSHGRI
NAVKGAVVYSNIVTTVSPTYALEVLSQGGRGLQDTLKVHSRKFLGILNGIDTDTWNPSTDRYLK
VQYNAKDLQGKAANKAALREQLNLASAYPSQPLVGCITRLVAQKGVHLIRHAIYKTAELGGQF
VLLGSSPVPEIQREFEGIADHFQDNNNIRLILKYDDALSHCIYAASDMFIVPSIFEPCGLTQMIAMR
YGSVPIVRKTGGLNDSVFDFDDETIPVEVRNGFTFVKADEQGLSSAMERAFNCYTRKPEVWKQL
VQKDMTIDFSWDTSASQYEDIYQKAVARARAAAHHHHHH

Third CBM53 domain of *Hv*SSIIIa:

TAVDVLYNPSNTVLNGKTEVWFRCSFNLWMHPSGALPPQKMVKSGDGSLLKATVNVPPDAYM
MDFVFSESDENGIYDNRNGMDYHIPVFPDIGQFYLNETSDLGLLPNPIPLGHHHHHH

Translated sequence of pET28a-SbeI(16-775)

MGSSHHHHHHSSGLVPRGSHMATAQDGADDLPIYDLDPKFAGFKDHFSYRMKKYRDQKHLIDE
HEGGLEEFSKGYLKFGINTENDATVYREWAPAAKEAQVIGDFNNWNGSGHRMTKDNFGVWSI
RISHVNGKPAIPHNSKVKFRFHRGDGVWVDRIPAWIRYAIADASKFGAPYDGVHWDPPTSERYV
FKHPRPQKPDAPRIYEAHVGMSGEKPEVSTYREFADNVLPRVKANNYNTVQLMAIKEHSYYASF
GYHVTNFFAASSRSGTPEDLKYLVDKAHSLGLRVLMDVVHSHASSNMTDGLNGYDVGQNTQE
SYFHTGERGYHKLWDSRLFNYANWEVLRFLLSNLRYWMDEFMFDGFRFDGVTSMLYNHHGIN
MSFSGDYKEYFGLDTDVDAVVYMMLANHLMHKLLPEATIVAEDVSGMPVLCRSVDEGGVGFD
YRLAMAIPDRWIGYLKNKDDLEWSMSGIAHTLTNRRYTEKCIAYAESHDQSIVGDKTMAFLLM
DKEMYTGMSDLQPASPTIDRGIALQKMIHFITMALGGDGYLNFMGNEFGHPEWIDFPREGNNWS
YDKCRRQWSLVDIDHLRYKYMNAFDQAMNALDDKFSFLSSSKQIVSDMNEEKKVIVFERGDLV
FVFNFHPNKTYDGYKVGCDLPGKYKVALDSDALMFGGHGRVAHDSDHFTSPEGIPGVPETNFN
NRPNSFKILSPPRTCVAYYRVEEKAEKPKDGGAAFWGKLLRDTSMLKLLASKDATDGEAISGSE
KVSTGDGSVKRGINFVFRSPDKDNK

Translated sequence of pET28a-SbeIIa(8-821)

MGSSHHHHHHSSGLVPRGSHMGATLGVARAAGGALPRSGSERRGGLDLPSLLLRKKDSSRAVL
SCAAAPGKVLVPDGESDDLAATPAQPEELQVPEDIEEEMAEVNMTGGAAEKLESSEPTQGIAETI
TDGVTKGVKELVVGEKPQVVPKPGDGQKIYEIDPTLKDFRSHLDYRYSEYKRIRAAIDQHEGGL
EVFSRGYEKLGFTRSAKGITYREWAPGAHSAALVGDFNNWNPNADTMTRDDYGVWEIFLPNN
ADGSPAIPHGSRVKIRMDTPSGVKDSISAWIKFSVQAPGEIPFNGIYYDPPEEEKYVFQHPQPKRP
ESLRIYESHIGMSSPEPKINSYANFRDEVLPRIKRLGYNAVQIMAIQEHSYYASFGYHVTNFFAPSS
RFGTPEDLKSLIDRAHELGLLVLMDIVHSHSSNNTLDGLNGFDGTDTHYFHGGPRGHHWMWDS
RLFNYGSWEVLRFLLSNARWWLEEYKFDGFRFDGVTSMMYTHHGLQMTFTGNYGEYFGFATD
VDAVVYLMLVNDLIHGLYPDAVSIGEDVSGMPTFCIPVPDGGVGFDYRLHMAVADKWIELLKQ
SDESWKMGDIVHTLTNRRWLEKCVTYAESHDQALVGDKTIAFWLMDKDMYDFMALDRPSTPR
IDRGIALHKMIRLVTMGLGGEGYLNFMGNEFGHPEWIDFPRGPQTLPTGKVLPGNNNSYDKCRR
RFDLGDADFLRYRGMQEFDQAMQHLEEKYGFMTSEHQYVSRKHEEDKVIIFERGDLVFVFNFH
WSNSFFDYRVGCSKPGKYKVALDSDDALFGGFSRLDHDVDYFTTEHPHDNRPRSFSVYTPSRTA
VVYALTE

Details on the expression and purification of all enzymes.

The cell growth and purification steps are as described in[17], with modifications as follows:

*Hv*GBSSI was purified with the IMAC step followed by dialysis against 20 mM Tris/HCl, pH 8.0, 150 mM NaCl, 10% (v/v) glycerol, 1 mM DTT and 1 mM EDTA.

For *Hv*SSI, both full length and truncated, the purification included only the IMAC and ion-exchange steps.

*Hv*SSIIa was purified with a modified IMAC step which included 1M urea in both buffer A and buffer B; and then either further purified by SEC, into a final buffer composed of 20 mM Tris/HCl, pH 8.0, 150 mM NaCl. 10% (v/v) glycerol, 1 mM DTT; or simply buffer exchanged via dialysis into a final buffer consisting of 20 mM Tris-HCl, 50 mM NaCl, 10% glycerol and 1 M Urea (pH 7.5). In the second case the urea is sufficiently diluted during the activity assays to have a negligible effect.

*Hv*SSIIIa and *Hv*SSIIIb were purified as for SSIIa, with SEC being the final step.

The third CBM53 domain of HvSSIIIa was expressed in ArcticExpress cells (Agilent Technologies) with induction for 16 h at 12°C. Purification was by IMAC at pH 7.5 in the presence of 300 mM NaCl, with no DTT or EDTA added. Protein fractions were pooled and used directly as they eluted from the column.

*Hv*SSIV was purified by IMAC at pH 8.0 in the presence of 250 mM NaCl, 1mM DTT. Protein fraction were pooled and subjected to IE after which SEC was employed.

Cloning, expression and purification of CBM20 from Aspergillus niger

The CBM20 encoding gene fragment from *Aspergillus niger* (327bp) was synthetically made and subcloned into pET-28a(+) (Invitrogen) between the NcoI and HindIII cloning sites. The resulting construct is CBM20 with with an N-terminal 6xHis-tag. The plasmid was amplified in *E. coli* strain DH5α (F– Φ80lacZΔM15 Δ(lacZYA-argF) U169 recA1 endA1 hsdR17 (rK–, mK+) phoA supE44 λ– thi-1 gyrA96 relA1). The protein was expressed in *E. coli* BL21(DE3) (F– ompT hsdSB(rB–, mB–) gal dcm (DE3)) in LB medium; containing 50 µg/mL kanamycin, at 37˚C and 250 rpm, to OD600 = 0.6. Expression was induced by adding isopropyl b-D-thiogalactopyranoside (IPTG) to 1 mg/ml, followed by incubation at 20˚C for 20 h. CBM20 was purified by standard His purification procedure.

List of acceptor substrates used in the activity assays:

Glucose was purchased from Merck (108342); maltose (M5895), maltotriose (M8378), maltotetraose (M8253), maltopentaose (M8128), maltohexaose (M9153) and maltoheptaose (M7753) were purchased from Sigma; maltooctaose from Carbosynth (OM06941). Glycogen was from Sigma (Sigma G8876, rabbit liver type III), as were soluble potato starch (S2630), maize amylopectin (10120) and potato amylopectin (A8515). Branched or alternative acceptor substrates substrates were purchased as follows: Panose from Sigma (P2407), 6^3-α-D-Glucosyl-maltotriose from Megazyme (O-GMT), 6^3-α-D-Glucosyl-maltotriosyl-maltotriose from Megazyme (O-GMH), Isomaltose from Sigma (I7253), Sucrose from Merck (7653), Maltosyltrehalose from Carbosynth (OM0698001101), Panose (P2407), maltosyl maltotriose (was a gift from Novozymes), maltosyl-β-cyclodextrin was purchased from Sigma (M9672).

Materials and methods for the thermostability spectrophotometric activity assay.

The enzymes were diluted in 10% glycerol, 250 mM NaCl, 20 mM Tris pH 8.5 and 1 mM DTT to a final concentration of 0.5 mg/mL, incubated for 15 minutes in a PCR tube in a PCR machine with the lid kept at 55°C or 60°C to minimize evaporation, then incubated in ice for five minutes, mixed and used after an appropriate dilution (only if necessary) in the coupled activity assay at 30°C. 10 mM maltopentaose was used as the acceptor substrate. In the case of GBSSI the enzyme was incubated at 3.0 mg/mL and used without further dilution, while SSI was incubated at 0.5 mg/mL, SSIIa was incubated at 0.12 mg/mL, SSIIIb was incubated at 0.1 mg/mL and SSIV was incubated at 0.05 mg/mL. The residual activity profiles were fitted to a sigmoidal curve with GraphPad Prism (GraphPad Software, San Diego California USA).

Materials and methods for the Thermofluor temperature stability assay.

Thermostability of *Hv*SSI was tested with a variant of the Thermofluor assay. *Hv*SSI was diluted to 0.1 or 0.2 mg/mL in buffer containing 20 to 125 mM Tris pH 8.0, 0 or 100 mM NaCl and 1X to 2.5X Sypro orange dye (Sigma-Aldrich #S5692). Different components were added to this buffer as needed. 50 µL of this solution were placed in individual wells of RT-PCR plates (MicroAmp® Optical 96-well reaction plate from Applied Biosystems, #4306737), sealed with adhesive film and centrifuged to remove bubbles and to create a flat surface. The plates were loaded into a 7500 RT-PCR system (Applied Biosystems) and subjected to a modified ramp protocol heating from 25°C to 95°C over 73 minutes (approx. 1°C/min). Fluorescence was monitored with ROX filters and the minimum points of its derivative were read manually and interpreted as melting temperatures Tm.

Thermostabilities mesured with the thermofluor assay.

Results with 0.1 mg/ml *Hv*SSI and 1X Sypro orange (20mM Tris pH 8.0, 100 mM NaCl):

Additive:	Tm	Δ Tm to reference
No additives	46.2°C	n/a
100 mM glucose	47.2°C	+1.0°C
10 mM maltose	46.5°C	+0.3°C
10 mM maltotriose	46.7°C	+0.5°C
10 mM maltopentaose	47.5°C	+1.3°C
10 mM maltooctaose	48.2°C	+2.0°C
0.1 mg/mL glycogen	46.2°C	+0.2°C
0.1 mg/mL soluble starch	46.1°C	-0.1°C
10 mM acarbose	46.6°C	+0.4°C
1 mM TCEP	46.4°C	+0.2°C
10% glycerol	49.3°C	+3.1°C
0.5 M NaCl	44.9°C	-1.3°C
5% glycerol + 250 mM NaCl	46.8°C	+0.6°C
4% PEG 3350	46.0	-0.2°C

Results for selected additives with 0.2 mg/mL *Hv*SSI and 2X Sypro orange (100 mM Tris pH 8.0, no NaCl):

Additive:	Tm	Δ Tm to reference
No additives	46.2°C	n/a
20 mM $MgCl_2$	45.3°C	-0.9°C
20 mM $CaCl_2$	44.4°C	-1.8°C
20 mM $SrCl_2$	45.0°C	-1.2°C
20 mM $BaCl_2$	44.8°C	-1.4°C
0.2 M LiCl	44.9°C	-1.3°C
0.4 M NaCl	45.0°C	-1.2°C
6% DMSO	45.0°C	-1.2°C
6% MPD*	40.1°C	-6.1°C
10% PEG400	45.1°C	-1.1°C
0.2 M glycine	47.2°C	+1.0°C
6% glucose	48.8°C	+2.6°C
6% sucrose	48.5°C	+2.3°C
6% xylitol	48.8°C	+2.6°C
6% sorbitol	48.4°C	+2.2°C
6% trehalose	47.7°C	+1.5°C
6% galactose	48.4°C	+2.2°C

Titrations with selected additives with 0.2 mg/mL *Hv*SSI, 2X Sypro orange, 20 mM Tris pH 8.0 and no NaCl:

Additive:	Tm	Δ Tm to reference
No additives	46.8°C	n/a
0.1 M urea	46.1°C	-0.7°C
0.2 M urea	45.7°C	-1.1°C
0.4 M urea	44.5°C	-2.3°C
0.7 M urea	42.7°C	-4.1°C
1.0 M urea	40.1°C	-6.7°C
1.5 M urea	35.9°C	-10.9°C
0.1 $(NH_4)_2SO_4$	46.6°C	-0.2°C
0.2 $(NH_4)_2SO_4$	46.3°C	-0.5°C
0.4 $(NH_4)_2SO_4$	45.4°C	-1.4°C
0.7 $(NH_4)_2SO_4$	no signal	
0.2 M NaCl	45.7°C	-1.1°C
0.5 M NaCl	44.9°C	-1.9°C
1.0 M NaCl	43.6°C	-3.2°C
1.5 M NaCl	41.2°C	-5.6°C
2.0 M NaCl	no signal	
5% glycerol	48.2°C	+1.4°C
10% glycerol	50.3°C	+3.5°C
10% glycerol + 10 mM DP8**	51.5°C	+4.7°C
10% glycerol + 20 mM DP8**	52.2°C	+5.4°C
10% glycerol + 40 mM DP8**	53.0°C	+6.2°C
20% glycerol	53.1°C	+6.3°C
30% glycerol	56.3°C	+9.5°C
5% MPD	41.5°C	-5.3°C
10% MPD	38.3°C	-8.5°C
15% MPD	35.5°C	-11.3°C

*MPD stands for 2-methyl-2,4-pentanediol

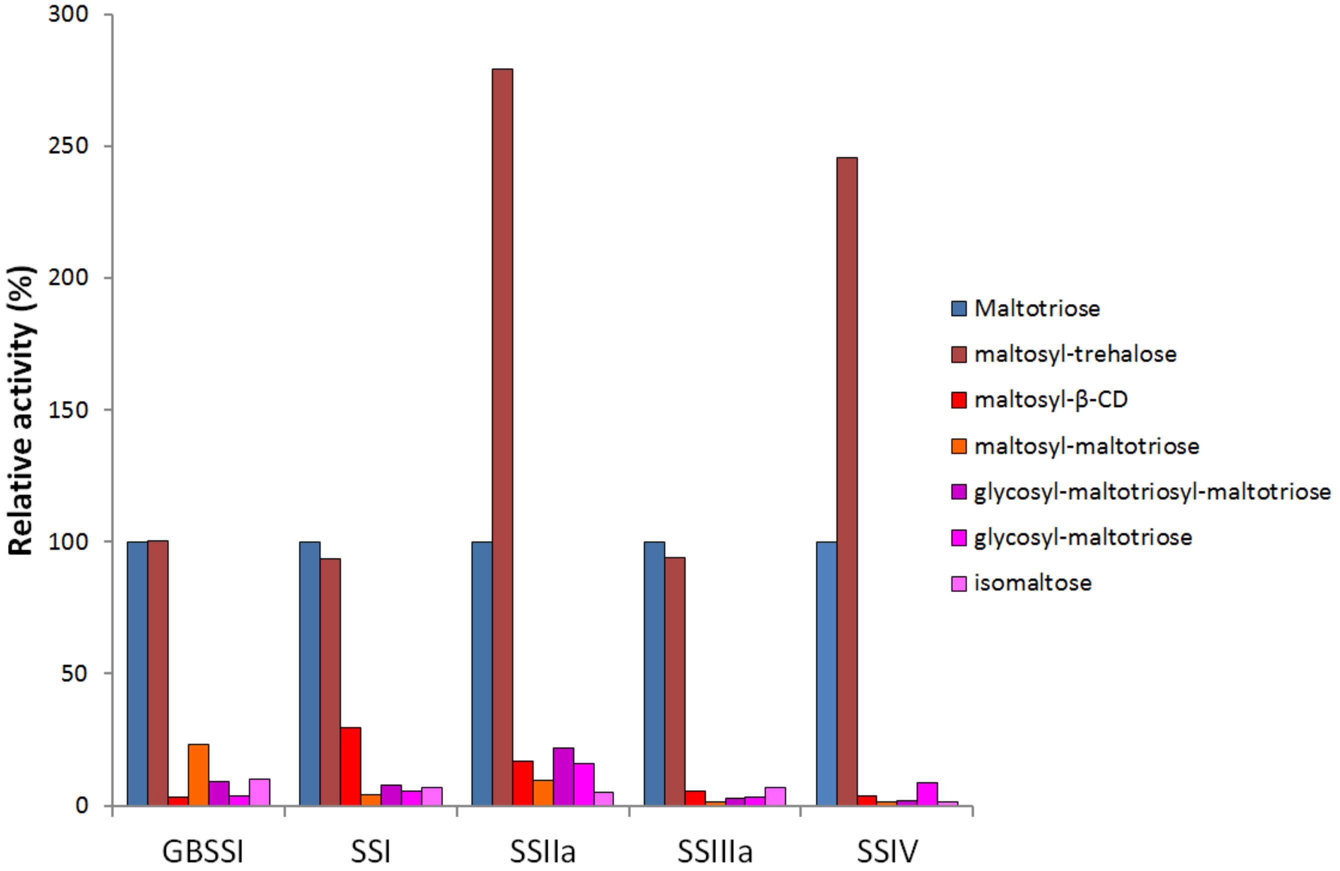

Figure S1: Activity of SS classes with other linear and branched oligosaccharide acceptors. Values are specific activities relative to maltotriose for each enzyme. Acceptors tested at 10 mM concentration except for maltosyl-maltotriose which was 1 mM, with 1 mM ADP-Glc at 37°C.

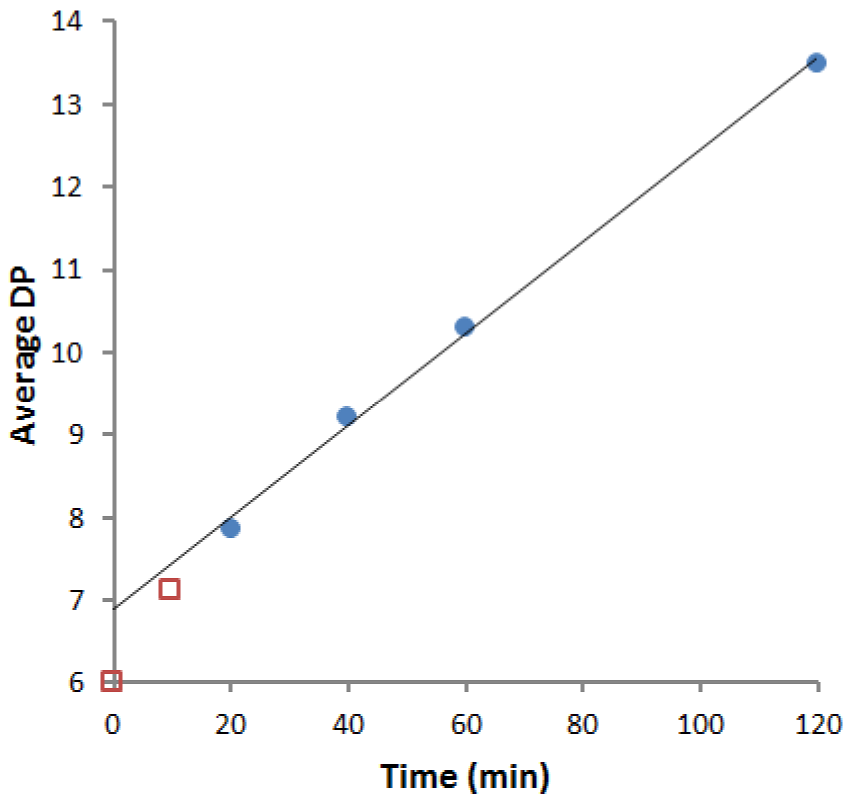

Figure S2: Progress of the reaction of SSI with fluorescently labeled acceptor between DP = 6 and DP = 13.5. The series depicts the average DP from integrals of the time series shown in Fig. 2c. The concentration of ADP-Glc can be considered constant and at saturating levels as only 4% of the initial 5 mM was consumed. The linear fit is for the four blue circles only and has a correlation coefficient of 0.998. The first two time points, depicted as open red squares, deviate from the linear trend, in agreement with the higher measured reaction velocities with maltohexaose and maltoheptaose as acceptors compared to maltooctaose or larger. Acceptors with DP = 6 and DP = 7 will still affect the data point at 20 minutes, even if to a lower extent than the first two. Removing this point from the linear fit produces a correlation coefficient of 1 for the three remaining data points.

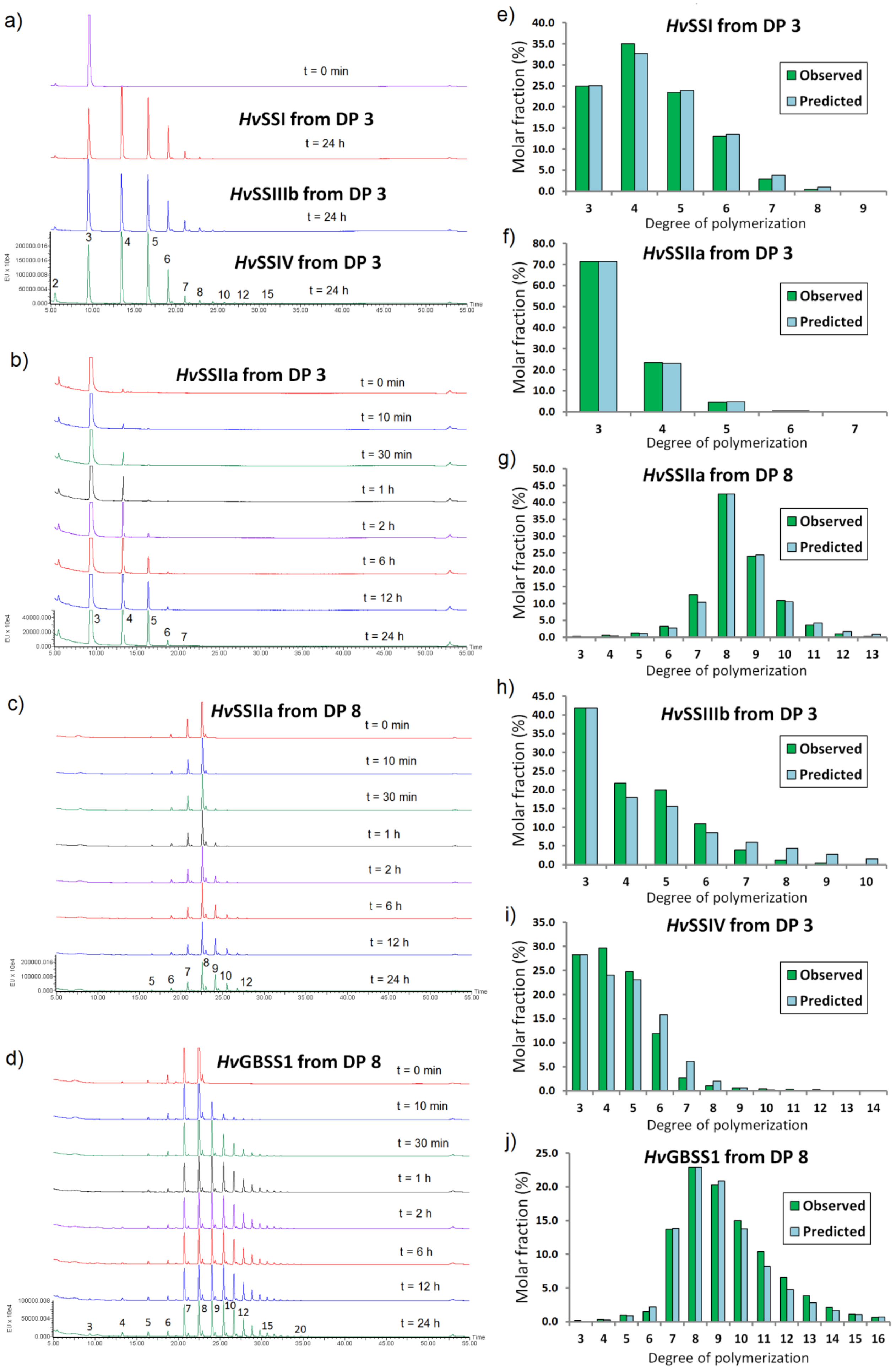

a)
t = 0 min
HvSSI from DP 3
t = 24 h
HvSSIIIb from DP 3
t = 24 h
HvSSIV from DP 3
t = 24 h
b)
HvSSIIa from DP 3
t = 0 min
t = 10 min
t = 30 min
t = 1 h
t = 2 h
t = 6 h
t = 12 h
t = 24 h
c)
HvSSIIa from DP 8
t = 0 min
t = 10 min
t = 30 min
t = 1 h
t = 2 h
t = 6 h
t = 12 h
t = 24 h
d)
HvGBSS1 from DP 8
t = 0 min
t = 10 min
t = 30 min
t = 1 h
t = 2 h
t = 6 h
t = 12 h
t = 24 h
e)
HvSSI from DP 3
f)
HvSSIIa from DP 3
g)
HvSSIIa from DP 8
h)
HvSSIIIb from DP 3
i)
HvSSIV from DP 3
j)
HvGBSS1 from DP 8
Observed
Predicted
Molar fraction (%)
Degree of polymerization

Figure S3: Estimation of processive or distributive mechanisms by comparison of observed and predicted product distributions. a), b), c), d), UPLC traces of reactions of the different SSs with unlabeled MOS. Labelling was done after reaction termination. For SSI, SSIIIb and SSIV only the products after 24 hours were analyzed, for GBSSI and SSIIa the time course of the reaction was followed. Within each panel the scale of the y-axis is the same and optimized for clarity of the smaller peaks. Peaks that were taller than the distance between traces have been truncated. DPs corresponding to each peak are indicated. e), f), g), h), i), j), observed product distributions corresponding to the relative peak areas in panels a-d (green bars) compared to the predicted product distributions simulated with purely distributive reaction models (blue bars). In each case, the time point in the simulation where the initially dominant peak had a molar fraction corresponding to the observed one was chosen, resulting in the perfect matches for DP 3 or DP 8 in each case. For SSI, SSIIa and SSIV, individual reaction velocities at 30°C were available and used, but for GBSSI and SSIIIb only velocities at 37°C were available and those were used in the simulations.

a) Donor kinetics

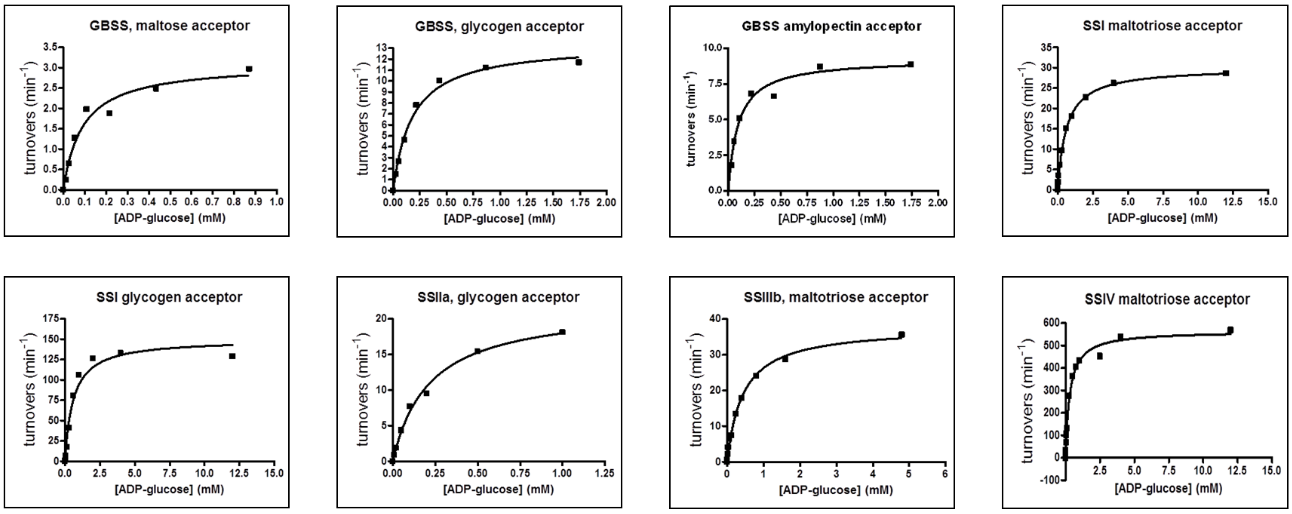

b) Glycogen acceptor kinetics

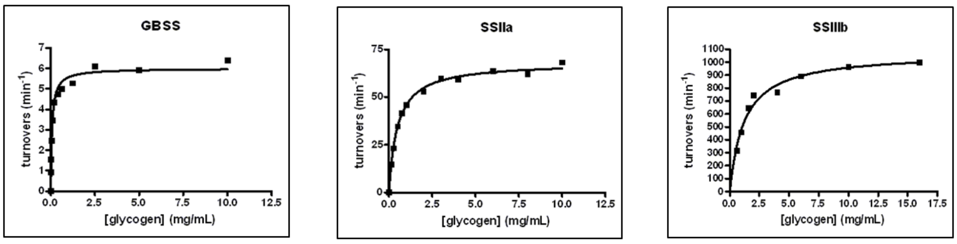

c) MOS acceptor kinetics

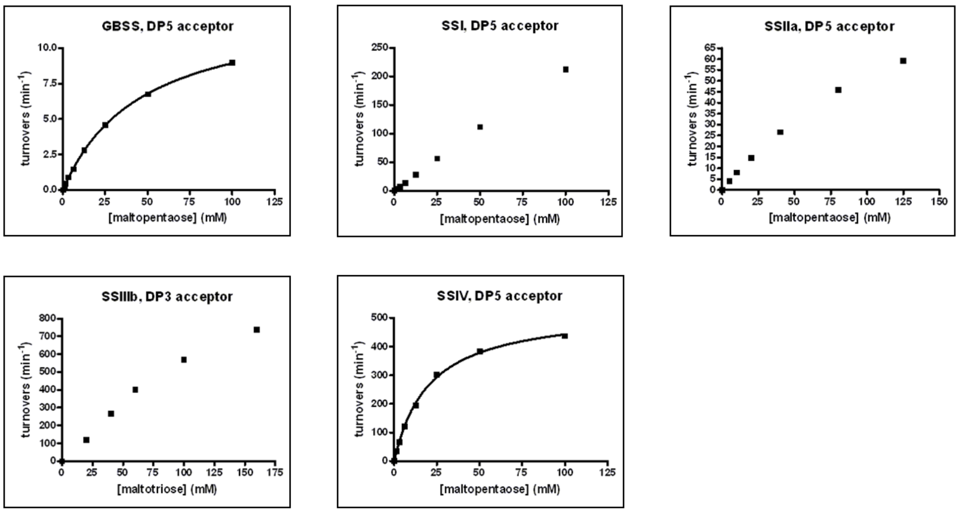

Fig S4: Michaelis-Menten fits of kinetic data. Michaelis-Menten curve fits for all instances where reliable parameters could be obtained by fitting the experimental data. In the absence of a curve fit, concentrations high enough to make a Michaelis-Menten fit could not be reliably achieved.

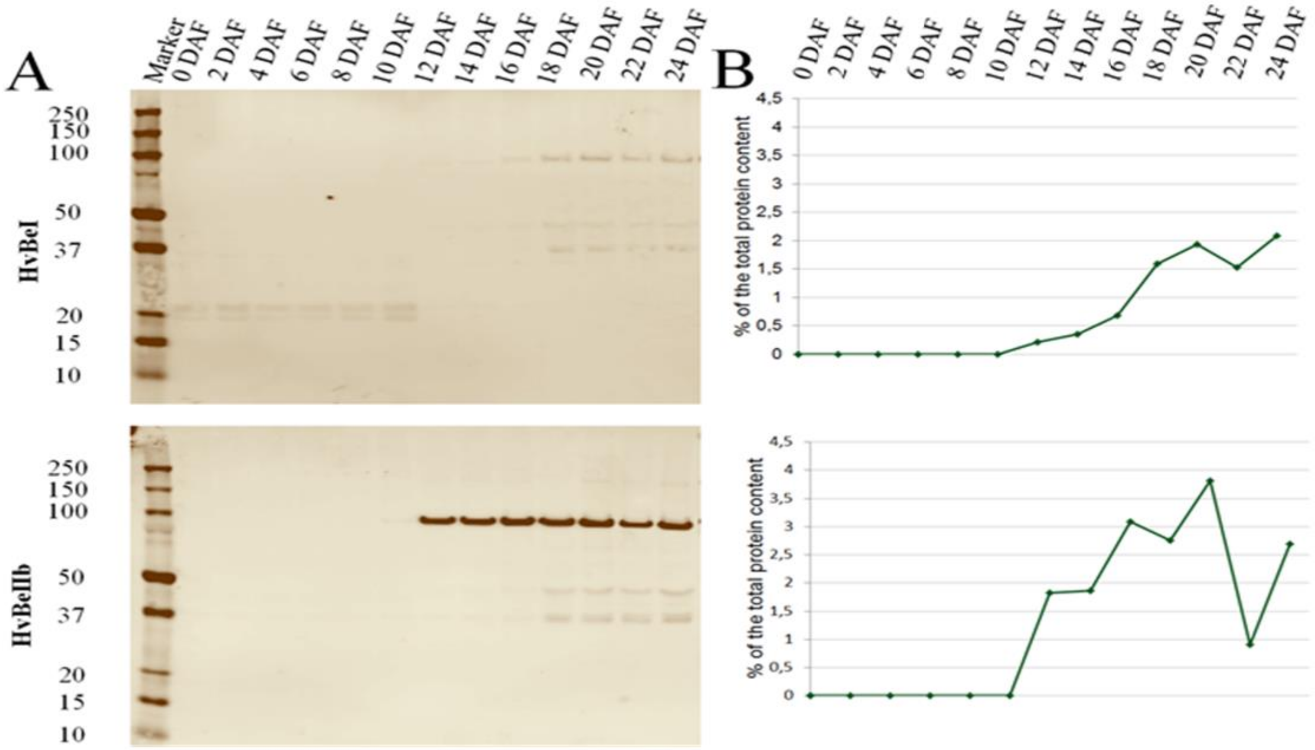

Figure S5: Temporal profile of protein abundance in endosperm, continued from main text. Barley grains were harvested between 0 and 24 DAF every two days and proteins from barley endosperm extracted. Equal amounts of protein were loaded onto SDS-PAGE and probed with the indicated primary antibodies. A) Immunoblots using indicated antibodies, protein ladder as indicated to the left of each immunoblot. B) Semi-quantitative analysis of protein abundance; x-axis showing time as DAF; y-axis showing protein quantity as calculated from immunoblot in percentage of total protein content. DAF, days after flowering; Be, branching enzyme.

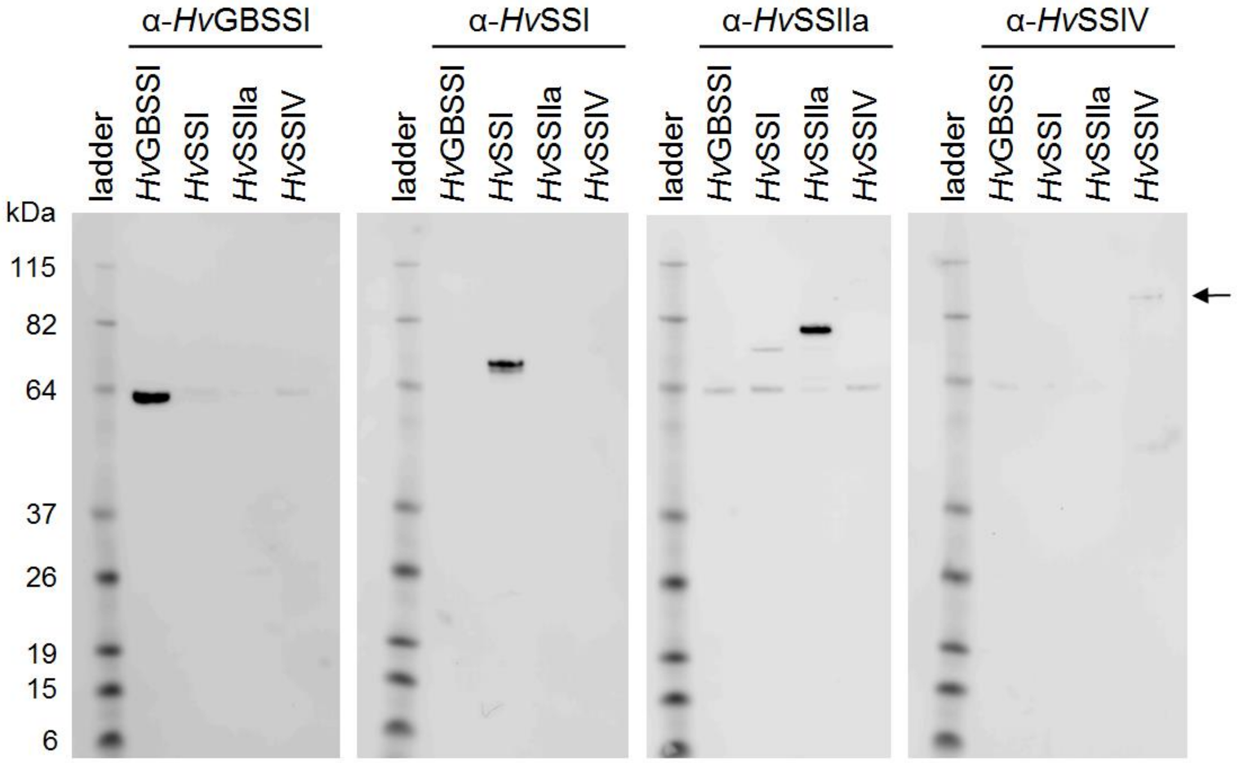

Figure S6: Specificity of the SS antibodies used. 10 ng per lane of recombinatly produced *Hv*GBSSI, *Hv*SSI, *Hv*SSIIa and *Hv*SSIV were loaded in gels in quadruplicate as indicated and transferred to immunoblots which were probed with each of the four anti-SS antibodies used in the main text. The antibodies used are indicated above each immunoblot. The molecular weights of the markers used in the ladder lanes are indicated. The molecular weights of the recombinant proteins used are: *Hv*GBSSI, 61.3 kDa; *Hv*SSI, 69.8 kDa; *Hv*SSIIa, 84.6 kDa and *Hv*SSIV, 99.1 kDa. Some degree of cross-reactivity is present and degradation to a lower level than in plant extracts is also visible is selected lanes. In all cases the main band is at the expected molecular weight and has intensity well in excess of any other bands in similar positions. The band for *Hv*SSIV, which is the same one used for quantification in Fig. 7, is indicated with a black arrow. Its intensity is much lower than that of comparable bands for the other SSs, an observation which was also made when preparing the calibration curves. It is likely that the affinity of this antibody is lower than in the other cases.
